# A nomogram model based on the number of examined lymph nodes–related signature to predict prognosis and guide clinical therapy in gastric cancer

**DOI:** 10.3389/fimmu.2022.947802

**Published:** 2022-11-02

**Authors:** Huling Li, Dandan Lin, Zhen Yu, Hui Li, Shi Zhao, Tuersun Hainisayimu, Lin Liu, Kai Wang

**Affiliations:** ^1^ School of Public Health, Xinjiang Medical University, Urumqi, China; ^2^ Department of Gastrointestinal Surgery, The Third Affiliated Hospital, Xinjiang Medical University, Urumqi, China; ^3^ Central Laboratory of Xinjiang Medical University, Urumqi, China; ^4^ JC School of Public Health and Primary Care, Chinese University of Hong Kong, Hong Kong, Hong Kong SAR, China; ^5^ Department of Biochemistry and Molecular Biology, Basic Medicine School, Xinjiang Medical University, Urumqi, China; ^6^ Department of Medical Engineering and Technology, Xinjiang Medical University, Urumqi, China

**Keywords:** gastric cancer (GC), the number of lymph nodes examined (ELNs), ELNs signature, nomogram, chemotherapy, immunotherapy

## Abstract

**Background:**

Increasing evidence suggests that the number of examined lymph nodes (ELNs) is strongly linked to the survivorship of gastric cancer (GC). The goal of this study was to assess the prognostic implications of the ELNs number and to construct an ELNs-based risk signature and nomogram model to predict overall survival (OS) characteristics in GC patients.

**Methods:**

This inception cohort study included 19,317 GC patients from the U.S. Surveillance, Epidemiology, and End Results (SEER) database, who were separated into a training group and an internal validation group. The nomogram was built with the training set, then internally verified with SEER data, and externally validated with two different data sets. Based on the RNA-seq data, ELNs-related DERNAs (DElncRNAs, DEmiRNAs, andDEmRNAs) and immune cells were identified. The LASSO–Cox regression analysis was utilized to construct ELNs-related DERNAs and immune cell prognostic signature in The Cancer Genome Atlas (TCGA) cohort. The OS of subgroups with high- and low-ELN signature was compared using the Kaplan–Meier (K-M) analysis. A nomogram was successfully constructed based on the ELNs signature and other clinical characteristics. The concordance index (C-index), calibration plot, receiver operating characteristic curve, and decision curve analysis (DCA) were all used to evaluate the nomogram model. The meta-analysis, the Gene Expression Profiling Interactive Analysis database, and reverse transcription–quantitative PCR (RT-qPCR) were utilized to validate the RNA expression or abundance of prognostic genes and immune cells between GC tissues and normal gastric tissues, respectively. Finally, we analyzed the correlations between immune checkpoints, chemotherapy drug sensitivity, and risk score.

**Results:**

The multivariate analysis revealed that the high ELNs improved OS compared with low ELNs (hazard ratio [HR] = 0.659, 95% confidence interval [CI]: 0.626–0.694, p < 0.0001). Using the training set, a nomogram incorporating ELNs was built and proven to have good calibration and discrimination (C-index [95% CI], 0.714 [0.710–0.718]), which was validated in the internal validation set (C-index [95% CI], 0.720 [0.714–0.726]), the TCGA set (C-index [95% CI], 0.693 [0.662–0.724]), and the Chinese set (C-index [95% CI], 0.750 [0.720–0.782]). An ELNs-related signature model based on ELNs group, regulatory T cells (Tregs), neutrophils, CDKN2B-AS1, H19, HOTTIP, LINC00643, MIR663AHG, TMEM236, ZNF705A, and hsa-miR-135a-5p was constructed by the LASSO–Cox regression analysis. The result showed that OS was remarkably lower in patients with high-ELNs signature compared with those with low-ELN signature (HR = 2.418, 95% CI: 1.804–3.241, p < 0.001). This signature performed well in predicting 1-, 3-, and 5-year survival (AUC [95% CI] = 0.688 [0.612–0.763], 0.744 [0.659–0.830], and 0.778 [0.647–0.909], respectively). The multivariate Cox analysis illustrated that the risk score was an independent predictor of survival for patients with GC. Moreover, the expression of prognostic genes (LINC00643, TMEM236, and hsa-miR-135a-5p) displayed differences between GC tissues and adjacent non-tumor tissues. The C-index of the nomogram that can be used to predict the OS of GC patients was 0.710 (95% CI: 0.663–0.753). Both the calibration plots and DCA showed that the nomogram has good predictive performance. Moreover, the signature was significantly correlated with the N stage and T stage. According to our analysis, GC patients in the low-ELN signature group may have a better immunotherapy response and OS outcome.

**Conclusions:**

We explored the prognostic role of ELNs in GC and successfully constructed an ELNs signature linked to the GC prognosis in TCGA. The findings manifested that the signature is a powerful predictive indicator for patients with GC. The signature might contain potential biomarkers for treatment response prediction for GC patients. Additionally, we identified a novel and robust nomogram combining the characteristics of ELNs and clinical factors for predicting 1-, 3-, and 5-year OS in GC patients, which will facilitate personalized survival prediction and aid clinical decision-making in GC patients.

## Introduction

Gastric cancer (GC) remains a major cancer globally, causing over a million new cases in 2020 and about 769,000 deaths, placing it fifth in incidence and fourth in mortality worldwide (1). In 2018, there are an estimated 456,124 new GC cases and 390,182 cases of GC-related death, which ranks third and second in cancer incidence rates and mortality rates, respectively, in China (2). The number of examined lymph nodes (ELNs) is regarded as the critical quality index for cancer care. The number of ELNs is essential as it guarantees adequate lymph node examination, improves the quality of pathology, and ensures the accuracy of lymph node staging (3, 4). Several studies have found that ELNs could reflect the extent of lymphadenectomy, and patients with more ELNs have improved prognoses (5–9). However, some studies have found that a positive correlation between ELNs and prognosis does not exist (10). In recent years, increasingly more researchers have been interested in the determination of optimal ELNs, and some studies advocated the minimum ELNs (8, 9, 11). However, the ideal number of retrieved lymph nodes remains unsettled, and the underlying mechanisms have not been elucidated.

For patients with GC undergoing radical total gastrectomy, the higher the ELNs, the better the prognosis, and the optimal threshold for ELNs is 21 or more (7). A study showed that both the Chinese and Surveillance, Epidemiology, and End Results (SEER) database populations were significantly associated with prognosis in patients with stage III GC after gastrectomy with systemic lymphadenectomy and recommended >31 ELNs to accurately assess the prognosis of GC patients (8). In ypN0 GC patients, ELNs were an independent predictor of survival. A minimum of 15 ELNs were recommended as the cutoff point for the evaluation of the quality of postoperative or prognostic stratification in ypN0 GC patients (9). These studies have shown that ELNs are related to the prognosis of GC, but the recommended number of ELNs is not the same. However, a recent GC study found that the multivariate Cox regression analysis showed that ELNs are no longer an independent prognostic factor of overall survival (OS) (10). To assess the relationship between ELNs and the OS of GC, the aforementioned studies are based on large numbers of people adjusted for age, sex, stage, and other basic characteristics. However, the mechanism through which ELNs improve survival time remains unclear. Thus, innovative strategies are needed to boost risk stratification and predict clinical outcomes with greater accuracy.

In contrast, little research has focused on revealing the molecular mechanisms underlying the different ELNs group in the genome. A comprehensive analysis of the link between genes, immune infiltration, and clinical prognosis is lacking for GC patients. Therefore, to understand the benefits of ELNs in predicting the prognosis of GC, we first used the GC data in the SEER database to find the optimal ELNs and evaluated the relationship between different ELNs subgroups and OS. Then, prognostic factors associated with GC were investigated, and a predictive nomogram was formulated for visualizing the information. On the basis of gene expression data and clinical data obtained from The Cancer Genome Atlas (TCGA) GC cohort, we developed an ELNs-related signature related to survival.

## Materials and methods

### Data set source and processing

#### Surveillance, epidemiology, and end results database

**Figure 1:**
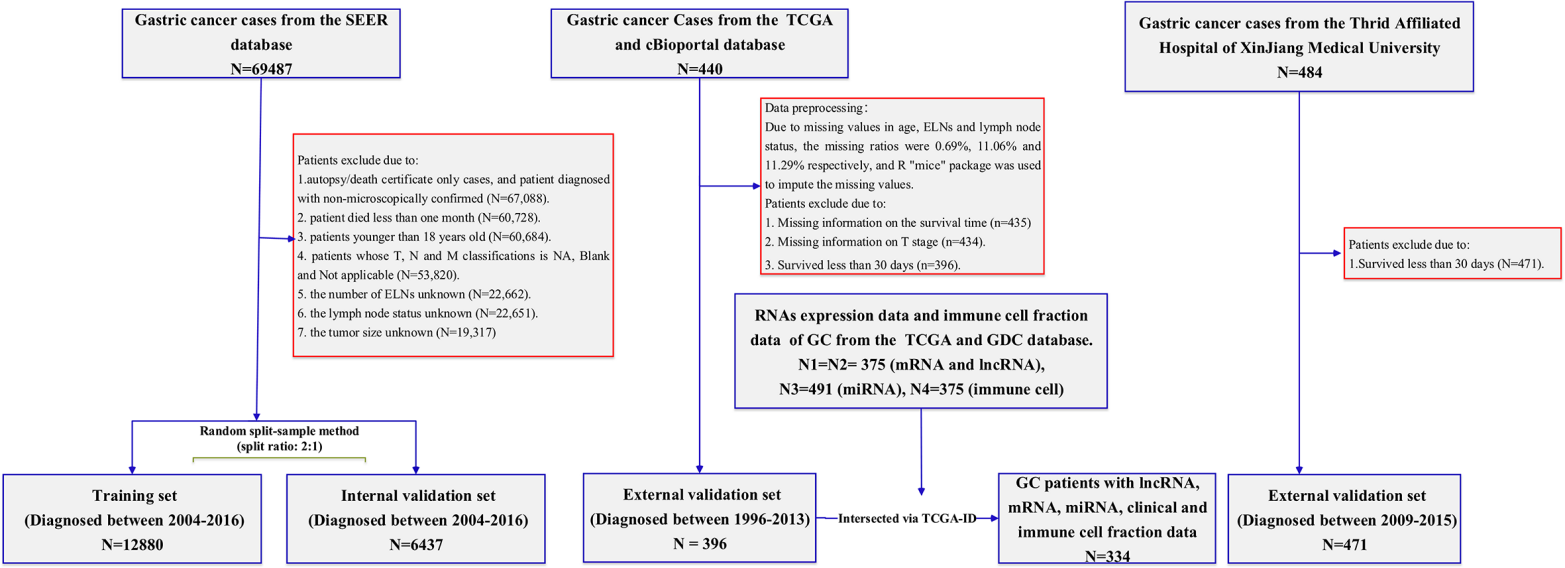
Flowchart illustrating gastric cancer patient selection for this study.

The SEER program (https://www.seer.cancer.gov) was initiated by the National Cancer Institute, which collects relevant information on patients in the United States from cancer registries. The largest geographic coverage of the database accounts for approximately 36.7% of the U.S. population. Data were extracted using the latest SEER*Stat software (version 8.3.9). The SEER*Stat database is Incidence-SEER 18 Regs Research Data, Nov. 2018 Sub (1975–2016). During the period 2004–2016, according to the International Classification of Disease for Oncology, Third Edition (histology code: ICD-O-3/WHO 2008), patients who pathologically confirmed stomach cancer as the first primary cancer were included in the research cohort for retrospective analysis and evaluation. The data selection process is shown in **Figure 1**. The inclusion criteria were as follows: (1) patient with microscopically confirmed diagnosis; (2) survival time of >1 month; (3) age of >18 years old; (4) and clinical and pathological characteristics including age at diagnosis, year at diagnosis, race, sex, marital status, grade, AJCC (American Joint Committee on Cancer) stage, TNM (Tumor Node Metastasis) status, regional nodes examined, regional nodes positive, tumor size, survival months, and vital status. We excluded patients with a diagnosis obtained exclusively from a death certificate or autopsy report, along with patients with regional nodes examined, regional nodes positive, and unknown tumor size.

Patients from 2004 to 2015 used the AJCC Staging Manual (sixth edition) to determine their pathological TNM status. Patients in 2016 used the SEER*RSA, a staging database developed by SEER, to determine the pathological TNM status. Regional nodes examined in SEER means the total number of regional lymph nodes removed and examined by the recording pathologist. Regional nodes positive in SEER means recording the exact number of regional lymph nodes that were found to have metastasis by the pathologist. The number of regional nodes positive is 0, which means that the lymph node status is negative, and the number of regional nodes greater than 0 means that the lymph node status is positive. The primary outcome of the study was OS, which was defined as the time from diagnosis to death or last follow-up. A receiver operating characteristic (ROC) curve was used to find the optimal cutoff of ELNs number to predict OS.

#### Chinese cohort

For the external validation set, Chinese patients from the Affiliated Tumor Hospital of Xinjiang Medical University diagnosed between 2009 and 2015 were used for external validation. The inclusion and exclusion criteria for the Chinese cohort were consistent with the SEER cohort. Demographic and clinicopathological data including age at diagnosis, gender, AJCC stage, grade, T stage, N stage, M stage, regional lymph nodes examined count, lymph nodes status, survival time, and survival status were collected. The institutional review committee of the participating institution approved the retrospective analysis of anonymous patient data. Since the study was retrospective, informed consent was not necessary, and patient data were used anonymously. A major outcome was OS, defined as the time from the date of diagnosis until death or last follow-up.

#### Other databases

The following databases were selected to obtain clinical information and omics data of GC patients:

1. TCGA (https://portal.gdc.cancer.gov/),
2. cBioPortal website (http://www.cbioportal.org/), and
3. Genomic Data Commons (GDC; https://cistrome.shinyapps.io/timer/).

The clinical characteristics of the GC patients were collected from the TCGA database and the cBioPortal website. The detailed clinical information included age, gender, race, grade, AJCC stage, T stage, N stage, M stage, regional lymph nodes examined count, lymph nodes status, survival time, and survival status. Patients with survival time of <30 days, unclear survival time, survival status, and clinical–pathological characteristics were excluded. The main outcome was OS, defined as the time from the date of diagnosis (diagnosed between 1996 and 2013) to the date of death or last follow-up. The gene expression RNA-seq (HTSeq-Count) and the miRNA expression RNA-seq (Illumina HiSeq) were obtained from the TCGA data portal. As this part of the data used in this study was downloaded from the TCGA database, following the TCGA’s strictly approved publication guidelines, there was no requirement for ethics committee approval. Moreover, immune infiltration information that consisted of every tumor specimen immune cell fraction of the 22 immune cell types was downloaded from the GDC. The specific processing flow of those cohorts is shown in **Figure 1**.

### Differentially expressed gene analysis

By using the “DESeq2” R package, we identified differentially expressed lncRNAs, miRNAs, and mRNAs between the high and low groups of ELNs. We selected DElncRNAs, DEmiRNAs, and DEmRNAs according to the same cutoff criteria: p < 0.05 and |log2 (foldchange)| > 0.5. The heat maps of differentially expressed lncRNAs, miRNAs, and mRNAs were generated by the function of the “ComplexHeatmap” R package.

### Construction of competing endogenous RNA network

The competing endogenous RNA (ceRNA) network was built using differentially expressed mRNAs, miRNAs, and lncRNAs. lncRNA–miRNA interaction information was predicted by the lncbase v.3 experimental module (http://carolina.imis.athena-innovation.gr/). The miRNA–mRNA interaction information was downloaded from miRTarBase databases (http://mirtarbase.cuhk.edu.cn/). The lncRNA–miRNA–mRNA coreRNA network based on the interactions between DElncRNAs and DEmiRNAs, as well as between DEmiRNAs and DEmRNAs, is created and visualized by using the “ggalluvial” R package.

### Immune cell scores

We used the Mann–Whitney *U*-test to identify significant differences in immune cell distributions of the high and low groups of ELNs. The R package “ggpubr” was used to draw the box plot.

### Development of the prognostic the number of lymph nodes examined signature

The genes and immune cells were transformed into binary variables, and the K-M curve analysis and the univariate Cox regression analysis were performed to screen prognostic genes and immune cells associated with patients’ OS. The genes and immune cells whose K-M and univariate Cox analyses’ p-value were <0.1 were inputted into the LASSO–Cox regression to identify the most useful predictive features. The ELNs group was also essential and included in the ELNs signature. The ELNs signature was calculated by the formula:

ELNs signature = ∑ Coefi * Vari where Coefi was the coefficient of each variable (ELNs group, gene, and cell) in the final Cox model, and Vari represented the variable value. For the ELNs group, the high-ELN group was given 1 point, and the low-ELN group was given 0 points. For each gene or cell, high expression was given 0 points, and low expression was given 1 point. According to the formula, all the GC patients in the TCGA cohort were separated into low- and high-ELN signature groups using the cutoff point calculated by the “survminer” package of R software. The K-M analysis was worked to measure the survival difference between the two risk subgroups.

### Validation of prognostic markers

Gene Expression Profiling Interactive Analysis (GEPIA; http://www.gepia.cancer-pku.cn/) is an interactive web server that analyzes RNA sequencing expression data across tumors and normal samples from TCGA and Genotypic Tissue Expression projects. The expression of each lncRNA and mRNA in normal tissues and cancer tissues can be obtained in the GEPIA database. Moreover, GC patients’ gene expression data and full clinical annotation were also searched in the GEO database. We systematically retrieved the databases with the key words “gastric cancer” and “survival.” There are some enrollment criteria as follows: data sets incorporating more than 30 human primary GC samples, series offered with OS time and survival status, and with transcriptome profiling as the experiment type. In total, seven eligible GC cohorts (GSE26253, GSE62254, GSE84437, GSE26899, GSE13861, GSE26901, and GSE28541) were gathered in this study for further analysis. Patients without survival information were not considered for further evaluation. The normalized matrix files for those cohorts were directly downloaded. The baseline clinical information of patients in all cohorts in this research is summarized (**Table S1**). Then, those cohorts were used to conduct a subsequent meta-analysis for prognostic markers. The combined value was calculated by the hazard ratio (HR) with a 95% confidence interval (CI). The χ^2^ and I^2^ statistical tests were applied to assess the heterogeneity between the involved data sets. If p > 0.05 or I^2^ < 50%, the fixed-effect model was used to calculate the combined effect. Otherwise, the random-effects model was used (p < 0.05 or I^2^ > 50%). The results display a series of forest plots created by the “forestplot” package of R software. For further evaluation of gene expression differences (mRNA) at the protein level, immunohistochemistry (IHC) staining images of gene protein expression in normal gastric tissues and gastric tumor tissues were acquired from the Human Protein Atlas (HPA; http://www.proteinatlas.org/) and analyzed.

### RNA extraction and reverse transcription–quantitative PCR analysis

#### Experimental specimens

In this study, 30 GC surgical specimens and paired normal adjacent tissues (normal tissues more than 5 cm away from the primary tumor) were selected from patients undergoing radical gastrectomy for GC in the Affiliated Tumor Hospital of Xinjiang Medical University from January 2018 to December 2020. All patients had signed informed consent before surgery, had no history of chemotherapy or radiotherapy before surgery, and were confirmed by pathological diagnosis after surgery. Fresh tumor tissues and normal adjacent tissues were immediately put into liquid nitrogen bottles for transfer and stored in a refrigerator at −80°C for RNA extraction.

#### Reverse transcription–quantitative PCR

Total RNA from tissues was extracted using a miRcute miRNA isolation kit (TIANGEN, Inc.). Total RNA was reverse-transcribed into cDNA using a FastKing RT kit (TIANGEN, Inc.) according to the manufacturer’s protocol. qPCR was subsequently performed on an ABI 7500 real-time PCR system (Applied Biosystems, Thermo Fisher Scientific, Inc.) with a SuperReal PreMix Plus (SYBR Green) reagent (TIANGEN, Inc.). qPCR was performed as follows: 95°C for 15 min, and 40 cycles of 95°C for 10 s and 60°C for 32 s. The primer sequences used for the qPCR are listed in **Table S2**. The expression levels of target genes were analyzed using the 2^−ΔΔCt^ method.

### Construction and evaluation of the nomogram model

First, we performed the univariate Cox regression analysis to evaluate the prognostic value of the ELNs signature and clinicopathological features. Subsequently, the multivariate Cox regression analysis was used to further determine the independent prognostic factors. A nomogram model construction was achieved by the “rms” package and the “survival” package in R. Finally, the concordance index (C-index), ROC curve, calibration curves, and decision curve analysis (DCA) were used to assess the consistency, accuracy, and clinical applicability of the nomogram model.

### Estimation of immune checkpoint expression

We assessed the expression levels of 50 immune checkpoints (ICPs) in GC samples in the TCGA cohort. The Wilcoxon rank-sum test was utilized to compare their expression difference in the high- and low-ELN signature.

### Drug sensitivity assessment

According to the public pharmacogenomics database Genomics of Drug Sensitivity in Cancer (GDSC; https://www.cancerrxgene.org), in the TCGA-STAD project, we calculated the half-maximal inhibitory concentration (IC_50_) of chemotherapeutic drugs using the R package to predict the response of GC patients to chemotherapy drugs. Using the Wilcoxon rank-sum test, we compared the difference in the estimated IC_50_ between the high- and low-risk groups.

### Statistical analyses

**Figure 2:**
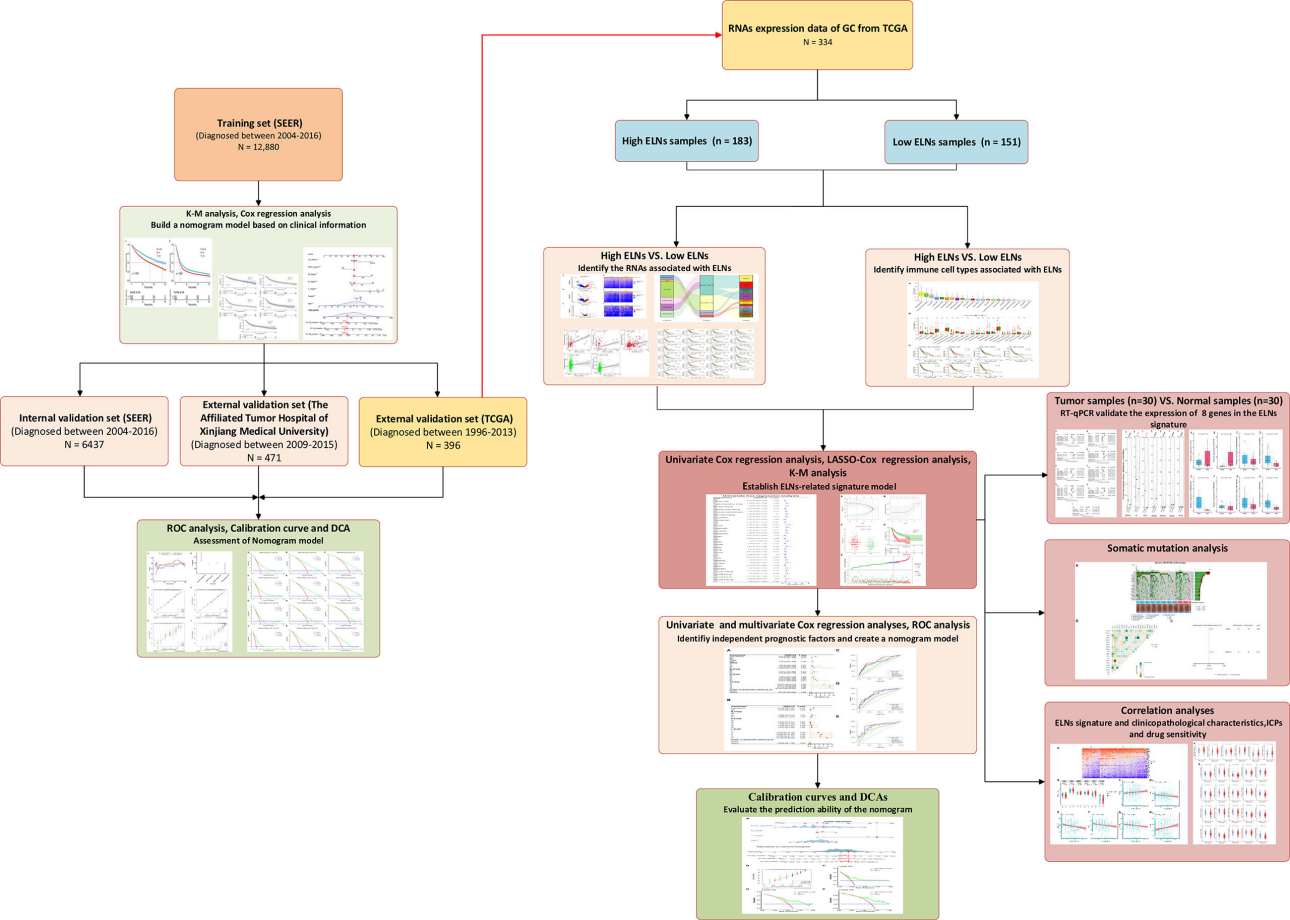
Flowchart of data analysis and experiment.

Categorical data and continuous data were shown as frequency and percentage, and mean and standard deviation, respectively, which were assessed by the χ^2^ test and the Mann–Whitney U-test, respectively. The paired t-test was used to test the significant difference between the paired samples. K-M survival analysis, univariate Cox regression analyses, LASSO regression, multivariate Cox regression analyses, ROC curve analysis, and DCA executed by the corresponding R packages were applied to the data sets. All optimal cutoff values (except for ELNs) were found using the “survminer” R package. The “mice” R package was used to perform multiple imputation procedures. The Spearman’s or Pearson’s test was used to conduct a correlation analysis between the two variables. R software (version 3.6.2, The R Foundation for Statistical Computing, https://www.r-project.org/) was used for all statistical analyses. Except for the special instructions, results with two-sided p-values of <0.05 were considered to be statistically significant. The detailed flow diagram of the study design is exhibited in **Figure 2**.

## Results

### Clinical characteristics analyses

#### Demographic and clinical characteristics in cohorts

The SEER database contained 19,317 GC patients, who were used as the model cohort. The random split sample method (split ratio 2:1) was used to divide the modeling cohort into training (12,880 cases) and internal validation cohorts (6,437 cases). In total, 62.9% of the patients were men, and 37.1% were women. The mean age of patients included was 65.69 years (18–101 years). The grade wears mostly III (62.3%); that is, the tumor characteristics showed a high proportion of poorly differentiated or undifferentiated cancers. The mean number of ELNs was 17.68, with 7,820 (40.5%) negative lymph nodes and 11,497 (59.5%) positive lymph nodes. In **Table S3**, you can find the demographics and clinicopathological characteristics of SEER’s training and internal validation cohorts, which were both comparable. A total of 396 patients were collected from the TCGA database and used as an external validation cohort for further mechanism analysis. There was also one external validation set, namely the Chinese validation set (n = 471). Demographic and clinicopathological characteristics of the two external validation cohorts are also listed in **Table S3**. The median follow-up time of the entire SEER data set and the internal validation data set were 24.00 months (interquartile range [IQR], 10.00–58.00 months) and 25.00 months (IQR, 10.00–57.00 months), respectively. Regarding the TCGA validation set and the Chinese validation set, they were 15.67 months (IQR, 9.49–26.44 months) and 24.63 months (IQR, 15.82–39.93 months), respectively. In addition, the 5-year OS of these data sets was also calculated. The findings displayed that, in the SEER training data set, the 5-year OS was 40.2% (95% CI, 39.3%–41.2%). For the SEER internal validation, TCGA, and Chinese external validation set, the 5-year OS values were 41.0% (95% CI: 39.6%–42.3%), 38.6% (95% CI: 30.5%–48.9%), and 62.0% (95% CI: 54.0%–71.2%), respectively.

#### Impact of the number of lymph nodes examined on survival

**Figure 3:**
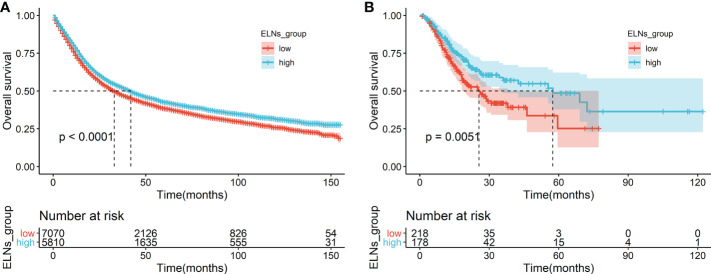
Survival analyses of OS in the ELNs group. **(A)** OS for high (>16) and low (≤16) ELNs in the training cohort. **(B)** OS for high (>16) and low (≤16) ELNs in the external validation cohort (The Cancer Genome Atlas). ELNs, the number of examined lymph nodes; OS, overall survival.

First, we analyzed the prognostic effect of ELNs in the training cohort. We determined the optimal cutoff value by the maximally selected rank statistics and divided the whole training cohort into two subgroups (low ELNs, ≤16; high ELNs, >16). Through the analysis of the clinical data of GC patients in the training cohort, we found that the high-ELN group and the low-ELN group were factors influencing the survival of GC patients. The K-M analysis revealed that the survival rate of the high-ELN group was better than that of the low-ELN group (p < 0.0001; **Figure 3A**). To explore the difference in gene expression levels between the two groups of patients with phenotype, we divided the GC patients in the TCGA database into the high-ELN group and the low-ELN group according to the cutoff value of the lymph node count obtained by the training cohort under the condition of ensuring that the phenotype is different. Consistent analysis results were also obtained in the clinical data of GC patients in the external validation cohort of the TCGA database (p < 0.01; **Figure 3B**).

In the training cohort, we further stratified by histological grade, N stage, and lymph node status and analyzed the prognostic effects of ELNs. A stratified analysis of histological grade showed (**Figures S1B, C**) that the OS of ELNs ≤ 16 and ELNs > 16 had a significant difference (p < 0.0001) in grades II and III. The survival benefit of the ELNs appeared to be stronger in N0–N2 (all p’s <0.0001; **Figures S2A–C**) compared with N3–NX (p = 0.27 and p = 0.60; **Figures S2D, E**). A stratified analysis by the lymph node status showed a statistically significant difference in the OS of patients between the ELNs ≤ 16 and ELNs > 16 groups (p < 0.0001; **Figures S3A, B**).

#### The construction of the STAD prognosis prediction model in the SEER database

**Figure 4:**
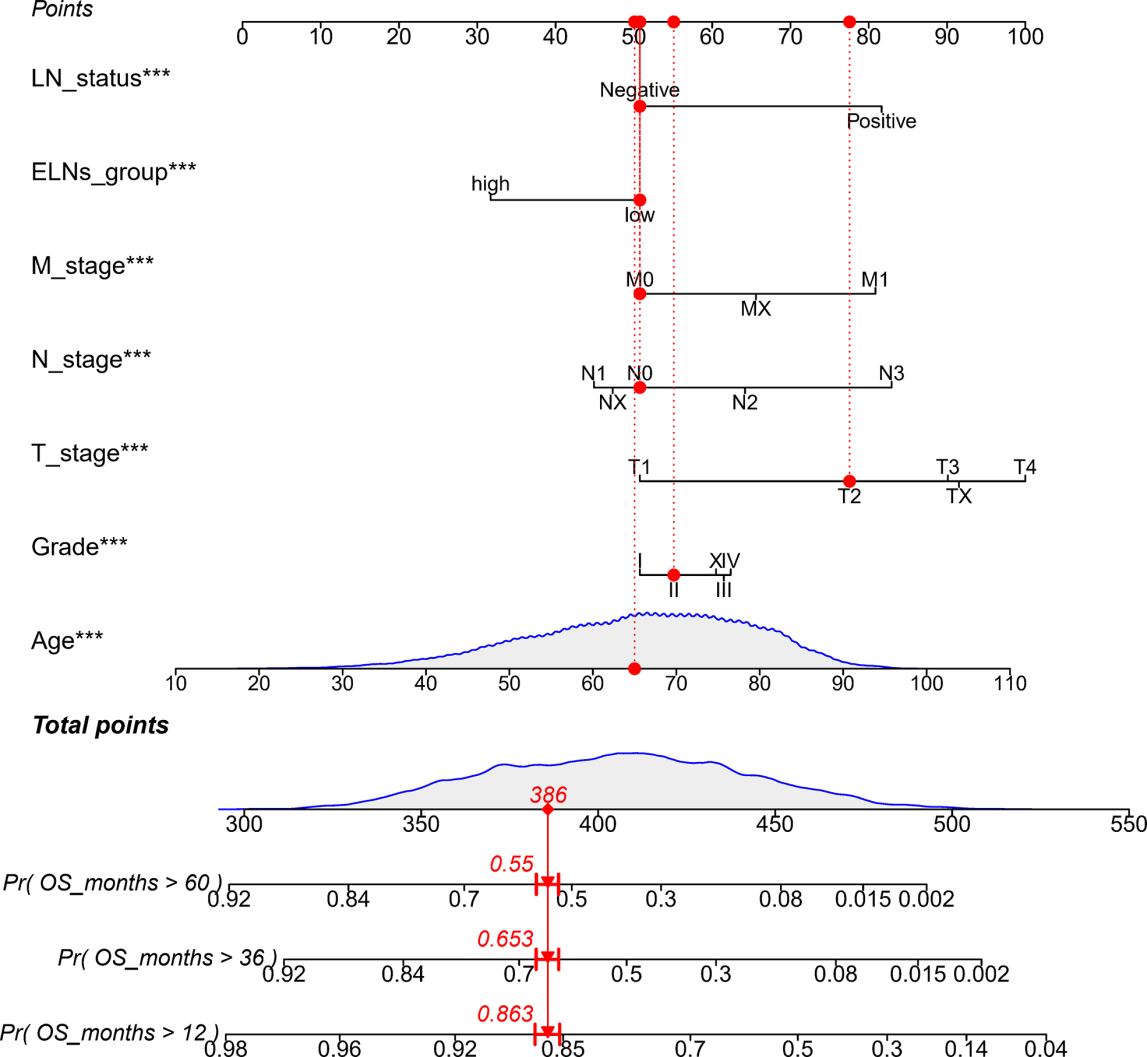
Nomogram to predict the OS of GC patients. ELNs, the number of examined lymph nodes; LN status, lymph node status; OS, overall survival; GC, gastric cancer. ***p < 0.001.

The results of the univariate Cox analysis showed (**Table S4**) that age, differentiation grade, T stage, N stage, M stage, ELNs group, lymph node status, and tumor size were all correlated with OS (all p’s < 0.05). The potential predictors (other than tumor size) identified in the univariate analysis were subsequently undertaken into the multivariate Cox analysis. Because the tumor size does not exist in the TCGA database and cannot be verified with the TCGA database, the tumor size is not included in the multivariate Cox analysis. The results demonstrated that age, differentiation grade, T stage, N stage, M stage, ELNs group, and lymph node status were all independent prognostic features of OS (all p’s < 0.05). Compared with low ELNs, patients with high ELNs had improved OS (HR = 0.659, 95% CI: 0.626–0.694, p < 0.0001). Higher ELNs were associated with better survival in GC, independent of age, differentiation grade, T stage, N stage, M stage, and lymph node status (**Table S4**). Based on the analysis results of the multivariate Cox of the training cohort, we described the influence of each variable on the risk of GC in the form of a nomogram. That is, a nomogram prediction model related to the occurrence of GC is established to predict the OS at 1, 3, and 5 years (**Figure 4**). In the nomogram, the first row is the score obtained for each variable. The sum of the scores of all variables in the model is the total score. The prediction corresponding to the total score vertically downward helped in estimating the 1-, 3-, and 5-year OS for each patient. Using 396 patients in the TCGA database and 471 patients in the Chinese cohort, we externally verified the nomogram and obtained similar analysis results.

#### Validating and comparing the predictive accuracy of the nomogram model in four data sets

**Figure 5:**
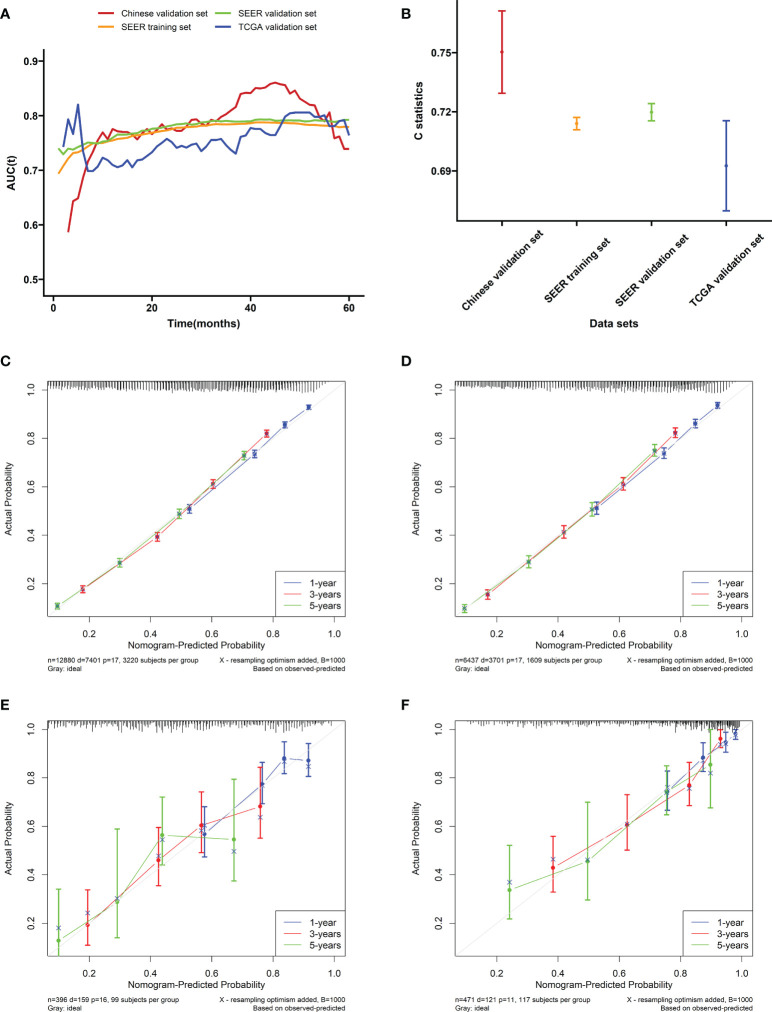
Evaluation of the nomogram. **(A)** The time-dependent AUC value of the nomogram in the training cohort (SEER), the internal validation cohort (SEER), the external validation cohort (TCGA), and the Chinese validation cohort. **(B)** The C-index of the nomogram in the training cohort (SEER), the internal validation cohort (SEER), the external validation cohort (TCGA), and the Chinese validation cohort. Calibration plots of the nomogram performed in the **(C)** SEER training, **(D)** the SEER internal validation, **(E)** the TCGA validation, and **(F)** the Chinese validation set, respectively.

**Figure 6:**
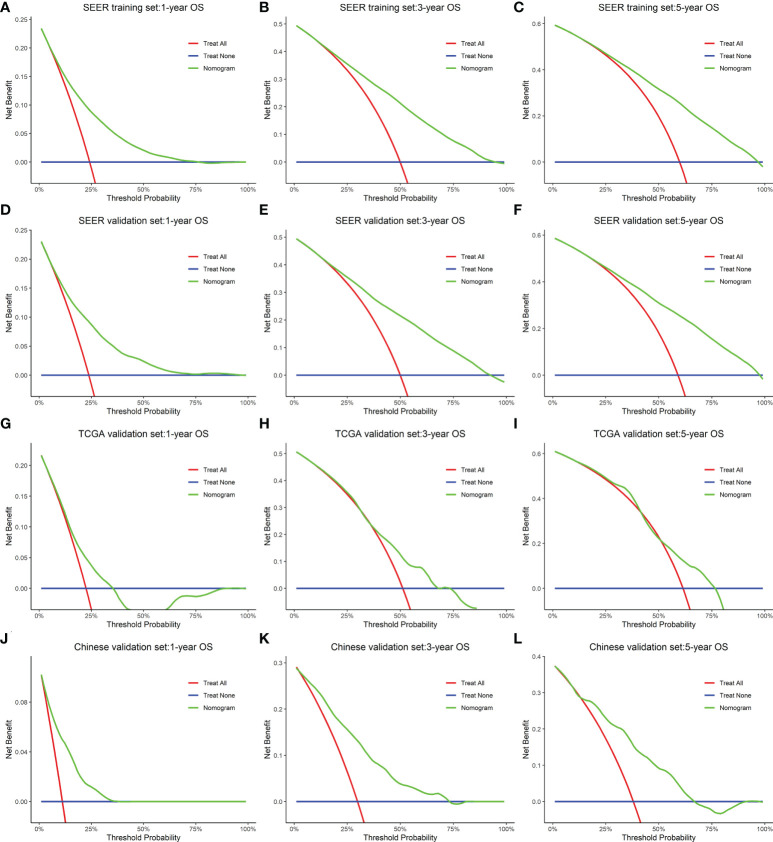
A decision curve analysis constructed for the nomogram that depicted the clinical net benefit for each cohort. **(A–C)** SEER training. **(D–F)** SEER internal validation. **(G–I)** TCGA validation. **(J–L)** Chinese validation set. As shown by the horizontal blue solid line, all patients are assumed not to be treated, whereas the solid red line indicates that all patients are treated. In all different cohorts, the nomogram provided superior net benefit across a range of threshold probabilities for decision curve analysis.

In the training cohort (SEER), the internal validation cohort (SEER), the external validation cohort (TCGA), and the Chinese validation cohort, the time-dependent AUC indicated that the nomogram model had a considerable value in predicting the OS in the GC cohort (**Figure 5A**). The AUCs of the nomogram predicting the 1-, 3-, and 5-year OS were 0.755, 0.784, and 0.779 in the training cohort (SEER); 0.762, 0.791, and 0.791 in the internal validation cohort (SEER); 0.665, 0.710, and 0.785 in the external validation cohort (TCGA); and 0.791, 0.815, and 0.762 in the Chinese validation cohort, respectively, indicating that the model has good prediction ability. The SEER internal validation data set confirmed excellent recognition capability of the nomogram (C-index [95% CI], 0.720 [0.714–0.726]). In addition, TCGA and Chinese external verification sets also confirmed this performance, with C-indices of 0.693 [0.662–0.724] and 0.750 [0.720–0.782], respectively (**Figure 5B**). The calibration plots of the 1-, 3-, and 5-year OS of the nomogram indicated that the predicted values of the training cohort (**Figure 5C**), the internal validation cohort (**Figure 5D**), the TCGA validation cohort (**Figure 5E**), and the Chinese validation cohort (**Figure 5F**) are in favorable agreement with the actual observations. Therefore, the constructed nomogram in this study performed well in both the training and validation sets. Additionally, DCA analysis was used to elucidate the net benefit at 1 (**Figures 6A, D, G**, **J**), 3 (**Figures 6B, E, H**, **K**), 5 (**Figures 6C, F, I**, **L**) years in four cohort. For instance, the net benefit ranges at 5 years in four cohort can be obtained. When the threshold probability of SEER training set and SEER internal verification set is between 24% and 98%, 52% and 76% in the TCGA validation set and 13% and 68% in the Chinese validation set, the usage of nomogram to predict the prognosis of GC patients offers a higher net benefit than the “all treat” or “no treat” strategies, indicating that the nomogram has good potential clinical applicability.

### Molecular features analyses of gastric cancer

By analyzing the clinical data of GC patients, our study showed that ELNs are independent prognostic factors for patients with GC, and the survival of the high-ELN group is better than that of the low-ELN group in the SEER database. The TCGA database also got consistent analysis results. To explore the differences at the molecular level between GC patients in the two groups (low-ELN and high-ELN groups), the 334 patients having both clinical information, miRNA, lncRNA, mRNA sequencing, and 22 immune cell fraction data were divided into high ELNs (n = 183) and low ELNs (n = 151) subgroups, according to the cutoff value of 16 ELNs from the SEER database.

#### Identification of differentially expressed lncRNAs, miRNAs, and mRNAs

**Figure 7:**
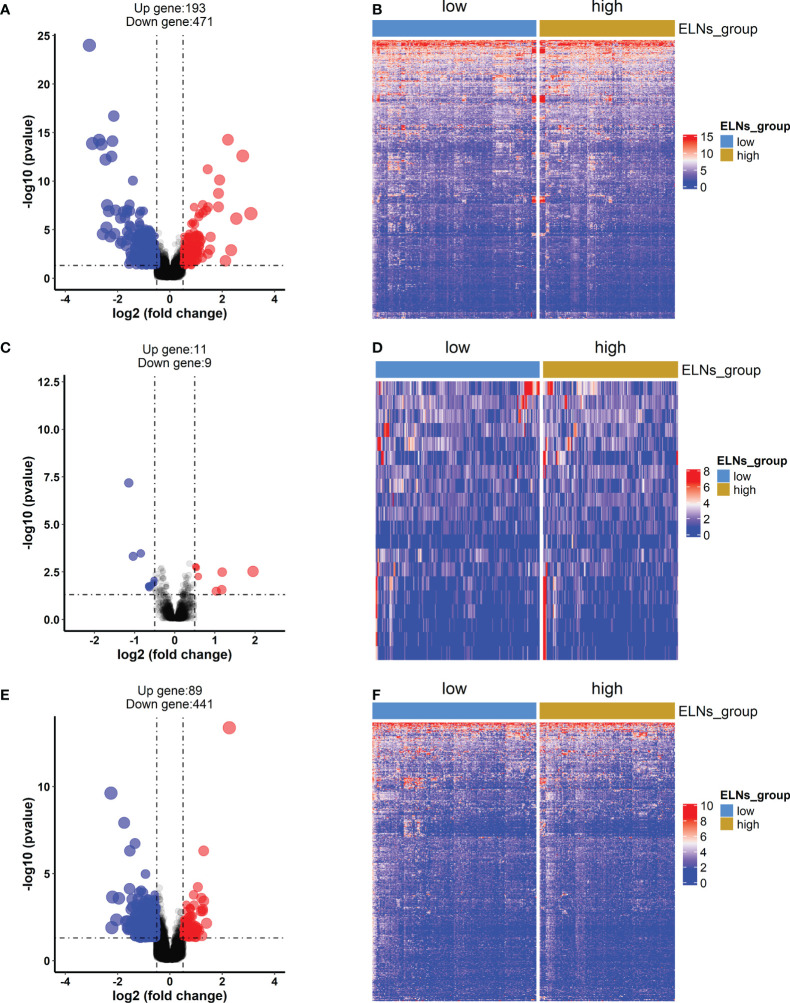
The differentially expressed mRNAs, lncRNAs, and miRNAs between the high-ELN and low-ELN groups were identified using the “DESeq2” package with R. The cutoff that we set was log2 (foldchange) > 0.5 or < −0.5 and p < 0.05. **(A, C, E)** The volcano plots of differentially expressed mRNAs (n = 664), lncRNAs (n = 530), and miRNAs (n = 20). Blue and red dots represent downregulated genes and upregulated genes, respectively. **(B, D, F)** Heat maps of the differentially expressed lncRNAs, miRNAs, and mRNAs between the high- and low-ELN groups.

First, the different expression analyses of miRNA, mRNA, and lncRNA were performed in the high- and low-ELN groups. The differentially expressed RNAs from the TCGA-STAD project were 14,333 lncRNAs, 2,055 miRNAs, and 19,568 mRNAs. Using the |log2 (foldchange)| > 0.5 and p < 0.05 as the cutoffs, we acquired 664 protein-coding genes (**Figures 7A, B**), 20 miRNAs (**Figures 7C, D**), and 530 lncRNAs (**Figures 7E, F**).

#### The construction of competing endogenous RNA networks and the survival analysis

**Figure 8:**
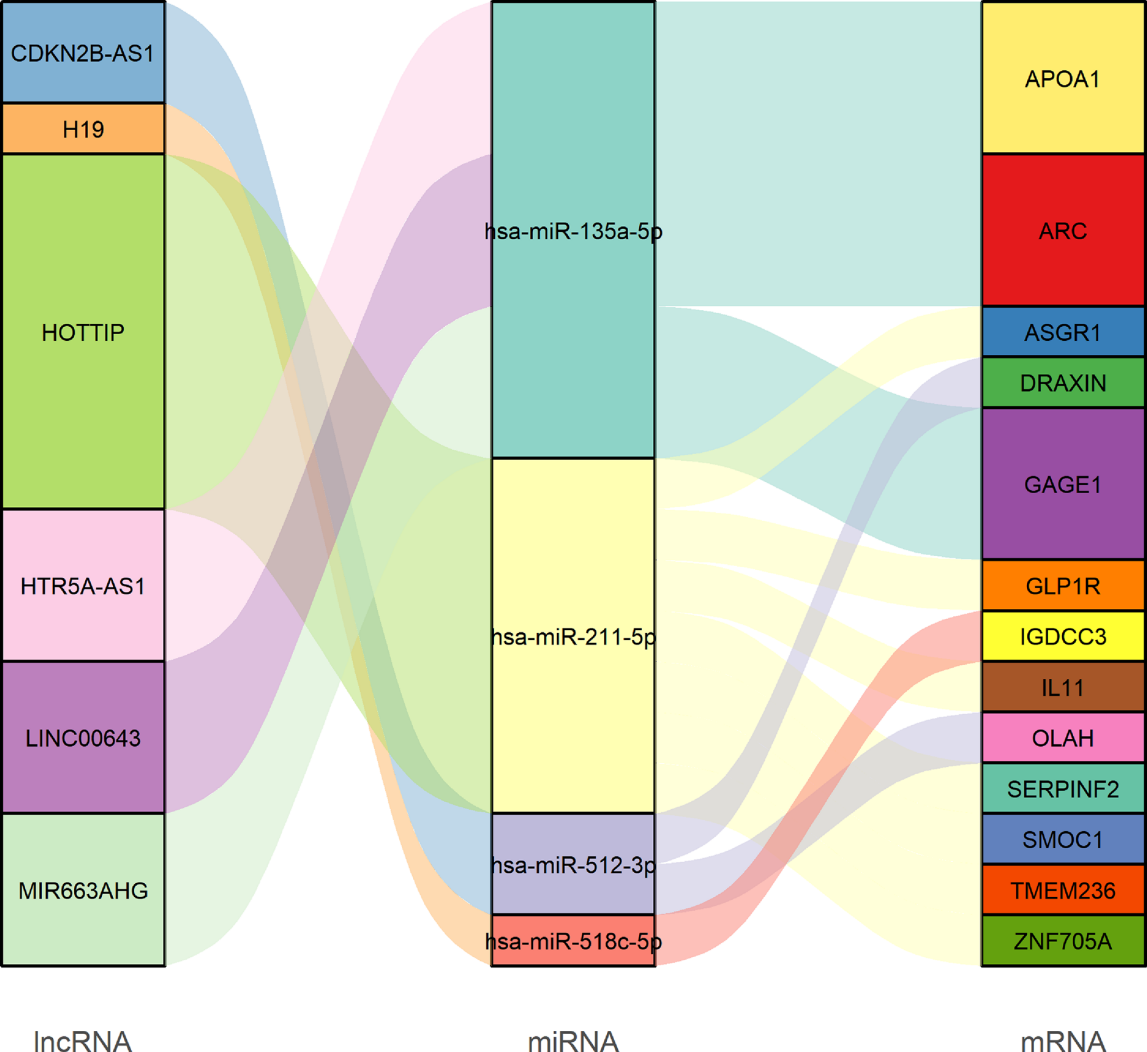
Sankey diagram of the competing endogenous RNA network in GC. Each rectangle represents a gene, and the connectedness of each gene is shown according to the size of the rectangle.

Next, a ceRNA network displaying the interactions between miRNAs, mRNAs, and lncRNAs was constructed based on the lncbase v.3 experimental module online tool and experimental verification from miRTarBase. According to the lncbase v.3 experimental module, target miRNA prediction revealed that six overlapped lncRNAs (eight lncRNA–miRNA links including six lncRNAs and six miRNAs) were obtained between 1,055 lncRNAs predicted from 20 miRNAs and the 530 differentially expressed lncRNAs. Target miRNA prediction revealed 35 miRNA–mRNA links composed of 14 miRNAs and 26 mRNAs according to the miRTarBase database. Finally, using R software, we constructed a STAD ceRNA regulatory network composed of 23 genes including 6 DElncRNAs, 4 DEmiRNAs, and 13 DEmRNAs (**Figure 8** and **Table S5**). **Table S6** provides detailed information about the ceRNA network. Moreover, we calculated the connection degree for genes related to the prognosis to understand their significance within the ceRNA network (**Figure 8** and **Table S5**). Among the lncRNAs, miRNAs, and mRNAs, HOTTIP (connection degree = 7), hsa-miR-135a-5p (connection degree = 9), APOA1, and ARC (connection degree = 3) are deemed the most significant. In the ceRNA network, hsa-miR-135a-5p had the highest connection degree (connection degree = 9), suggesting a strong impact on the pathogenesis of STAD.

lncRNAs are thought to interact directly with miRNAs to positively regulate mRNA expression, as explained by the ceRNA theory. To validate this phenomenon in STAD, we analyzed the correlation between DElncRNAs and DEmRNAs targeted by hsa-miR-135a-5p, which was a gene with the highest degree of connectivity within the ceRNA network. We detected positive correlations between DElncRNAs and DEmRNAs targeted by hsa-miR-135a-5p. **Figures S4A–C** showed the top three correlation coefficients of interactions, in which HTR5A-AS1 interacts with GAGE1 (R = 0.34, p = 9.57E−11), MIR663AHG interacts with GAGE1 (R = 0.39, p = 1.50E−13), and MIR663AHG interacts with APOA1 (R = 0.22, p = 5.03E−05). Moreover, we also verified the relationship between the lncRNAs and mRNAs in other dependent data sets (GEO data sets: GSE62254 and GSE84437). Among the correlation coefficients between DElncRNA and DEmRNA targeted by hsa-miR-135a-5p, GAGE1 and MIR663AHG had the highest correlation coefficient, so we only verified the relationship between GAGE1 and MIR663AHG. The correlation analysis results of these two data sets (GSE62254 and GSE84437) reveled weak positive correlations between GAGE1 and MIR663AHG, which are consistent with the results of TCGA (**Figures S4D–E**).

To obtain DERNAs closely associated with the prognosis of GC patients, we performed univariate Cox regression, K-M survival curve analysis, and log-rank test on each DERNA in the constructed ceRNA network. According to their respective optimal cutoff values, GC patients were categorized into high-expression and low-expression groups. As a result, we obtained 10 DERNAs (namely five DElncRNAs: MIR663AHG, LINC00643, HOTTIP, CDKN2B-AS1, and H19; one DEmiRNA: hsa-miR-135a-5p; and four DEmRNAs: APOA1, ARC, TMEM236, and ZNF705A), which were correlated with OS (all p’s < 0.1). Among these genes, two DElncRNAs and two DEmRNAs have a protective effect (HRs < 1) because patients with low expression levels of these RNAs have a better prognosis than patients with high expression levels. On the contrary, the remaining three DElncRNAs, one DEmiRNA, and two DEmRNAs were considered oncogenes (HRs > 1) because their expression is negatively correlated with the prognosis of GC patients. The survival curves and univariate Cox regression results of all DElncRNAs, DEmiRNAs, and DEmRNAs are displayed in **Figures S5**, **S6**.

#### Estimation of immune cell-type fractions in GC and the survival analysis

**Figure 9:**
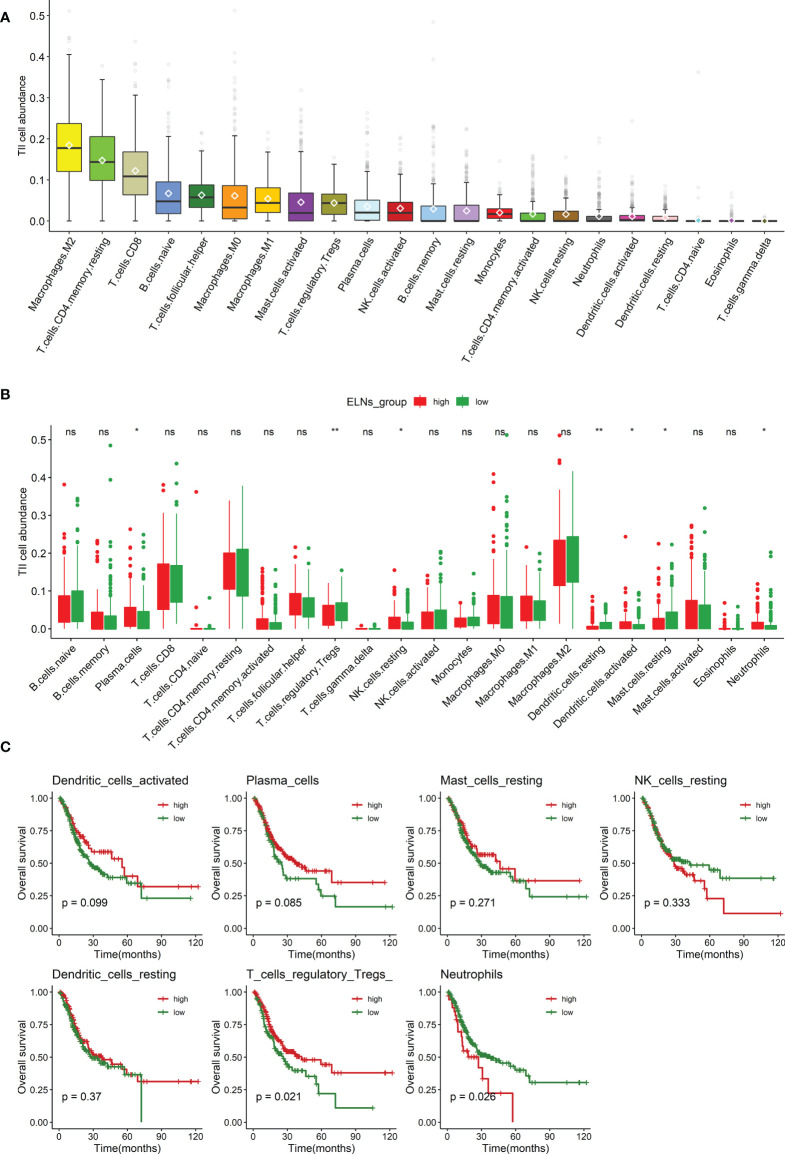
Analysis of the ELNs-related TIICs. **(A)** Distribution of 22 types of TIICs in gastric cancer. **(B)** Box plot displays the abundance differentiation of 22 types of immune cells between the GC samples with low- and high-ELN groups, and the significance test was carried out by the Wilcoxon rank-sum test. **(C)** Kaplan–Meier and log-rank test for seven immune cells passed the Wilcoxon rank-sum test. Four representative immune cells including plasma cells, neutrophils, and regulatory T cells (Tregs) are shown based on their respective optimal cutoff values (all p < 0.1). ELNs, the number of lymph nodes examined; TIICs, tumor-infiltrating immune cells; GC, gastric cancer. *p < 0.05; **p < 0.01.ns, no significance.

We measured the abundance of tumor-infiltrating immune cells (TIICs) in GC tissue using the CIBERSORT algorithm. The box plot (**Figure 9A**) could indicate that macrophages M2, T-cell CD4 memory resting, and T-cell CD8 were significantly high expression in the GC tissue, and they might play an essential role in GC. The results of the Wilcoxon–Mann–Whitney U-test suggested that the distribution of several immune cell fractions in the high-ELN group was different from that in the low-ELN group, including plasma cells, neutrophils, Tregs, NK cells resting, dendritic cells resting, dendritic cells activated, and mast cells resting (all p’s < 0.05; **Figure 9B**). To determine which immune cell has an impact on the OS of GC patients, we also conducted univariate Cox regression, K-M survival curve analysis, and log-rank test for seven immune cells that passed the Wilcoxon rank-sum test (**Figures 9C**, **S6**). Among these immune cells, neutrophils were positively associated with the prognosis of GC patients because patients with low expression levels of this cell have a longer prognosis than patients with high expression levels, which suggested the protective roles of this cell in GC development. On the contrary, plasma cells and Tregs were considered risk factors because their expression is negatively correlated with the prognosis of GC patients.

#### The composite and coexpression analysis of genes and tumor-infiltrating immune cells

**Figure 10:**
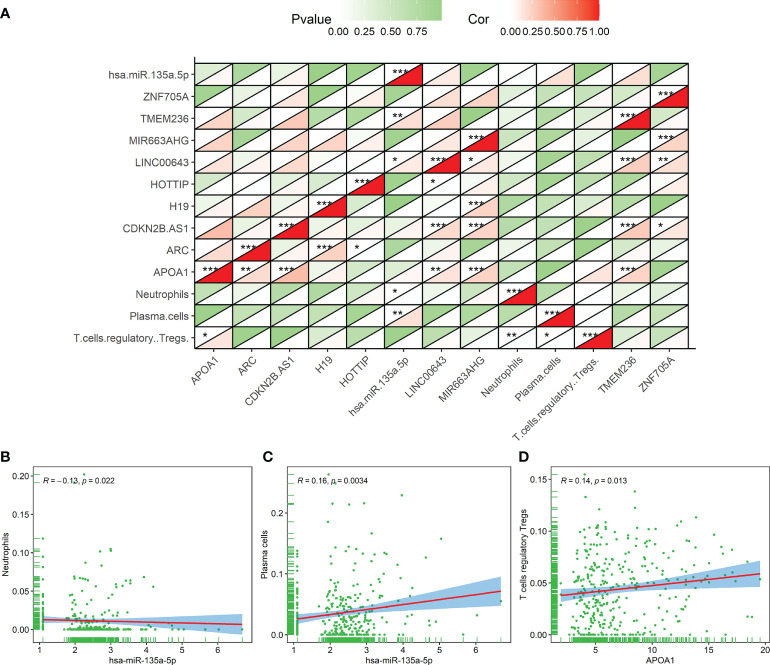
The correlation result of the coexpression analysis between tumor-infiltrating immune cells and DERNAs related to the prognosis of gastric cancer patients. **(A)** The coexpression heat map illustrated the coexpression patterns of 10 genes and 3 immune cells. **(B–D)** Neutrophils and hsa-miR-135a-5p (R = -0.13, p = 0.022), plasma cells and hsa-miR-135a-5p (R = 0.16, p = 0.0034), and regulatory T cells and APOA1 (R = 0.14, p =0.013). *p < 0.05; **p < 0.01; ***p < 0.001.

We further analyzed and illustrated the correlation between TIICs and DERNAs. Spearman analysis was used to demonstrate some significant coexpression patterns about 10 DERNAs and 3 immune cells associated with the prognosis of the GC patients (**Figure 10A**). The results revealed that neutrophils (**Figure 10B**) and plasma cells (**Figure 10C**) had a positive correlation with the hsa-miR-135a-5p expression (R = -0.13, p = 0.022; R = 0.16, p = 0.0034), and Tregs (**Figure 10D**) had a positive correlation with APOA1 expression (R = 0.14, p = 0.013). We could further verify that the expressions of hsa-miR-135a-5p and APOA1 significantly influenced the immune activity of the tumor microenvironment (TME) from the above outcomes.

#### Construction of the number of lymph nodes examined signature for overall survival

**Figure 11:**
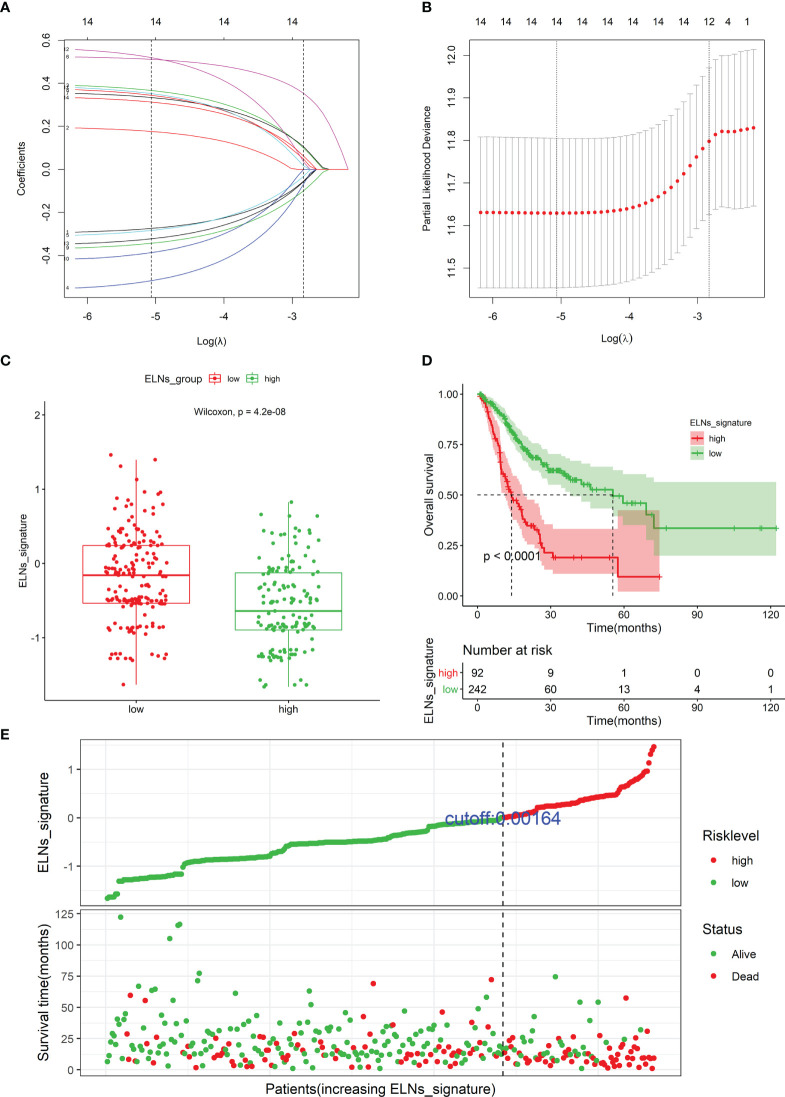
Identifying prognostic genes and cells for developing an ELNs signature. **(A)** LASSO coefficient profiles of the 14 survival-related factors in the TCGA cohort. **(B)** Selection of the optimal parameter (lambda.1se = 0.05892685) in the LASSO regression model. **(C)** The distribution of the ELNs signature between the high- and low-ELN groups using Mann–Whitney U-test. **(D)** Kaplan–Meier survival curve of patients with high- and low-ELN signature groups. **(E)** Distribution of the ELNs signature in the TCGA cohort. ELNs, the number of lymph nodes examined.

A total of 14 factors were significantly related to the OS of GC patients in this study. The result of the univariate Cox regression for the ELNs group, 10 genes (APOA1, ARC, TMEM236, ZNF705A, MIR663AHG, LINC00643, HOTTIP, CDKN2B-AS1, H19, and hsa-miR-135a-5p), and 3 immune cells (plasma cells, neutrophils, and Tregs) was illustrated by the forest plot in **Figure S6**. Next, to build an optimal prognostic ELNs signature for OS, we used the LASSO–Cox analysis to identify key prognostic indicators. The LASSO regression model was optimal when 12 variables with lambda.1se = 0.05892685 were selected as the target markers (**Figures 11A, B**). We applied the factors derived from the LASSO regression analysis to the multivariate Cox regression analysis to construct the optimal ELNs signature. Furthermore, the risk coefficients generated by the multivariate Cox regression analysis were used to calculate the ELNs signature of each patient. The formula of the ELNs signature was based on the corresponding coefficients of variables with p < 0.1 (**Table S7**):

ELNs signature = −0.35569 * ELNs group + 0.38558 * Tregs| CIBERSORT + −0.52900 * Neutrophils | CIBERSORT + −0.36941 * CDKN2B-AS1 | lncRNA + 0.55315 * H19 | lncRNA + 0.41669 * HOTTIP | lncRNA + 0.36619 * LINC00643 | lncRNA + −0.40853 * MIR663AHG | lncRNA + 0.49878 * TMEM236 | mRNA + −0.32665 * ZNF705A | mRNA + 0.35357 * hsa-miR-135a-5p | miRNA.

Among these factors in the ELNs signature, the ELNs group, neutrophils, CDKN2B-AS1, MIR663AHG, and ZNF705A were protective factors for GC patients’ OS, with HRs of <1, and Tregs, H19, HOTTIP, LINC00643, TMEM236, and hsa-miR-135a-5p were risk factors, with HRs of >1. Distributions of the ELNs signature revealed that patients in the high-ELN group had lower ELNs signature than patients in the low-ELN group (p < 0.001; **Figure 11C**). According to the optimal ELNs signature cutoff value (0.00164), all patients were divided into the high-ELN signature (n = 92) and low-ELN signature (n = 242) groups. The K-M survival curve showed that the patients with a low-ELN signature exhibited a longer survival time than those in the high-ELN signature group (log-rank test: p < 0.0001; **Figure 11D**). The ELNs signature and the survival status distribution of each case were shown in **Figure 11E**. Remarkably, the number of deaths was dramatically higher in the high-ELN signature group.

#### The verification of prognostic markers in the number of lymph nodes examined signature by database and reverse transcription–quantitative PCR

**Figure 12:**
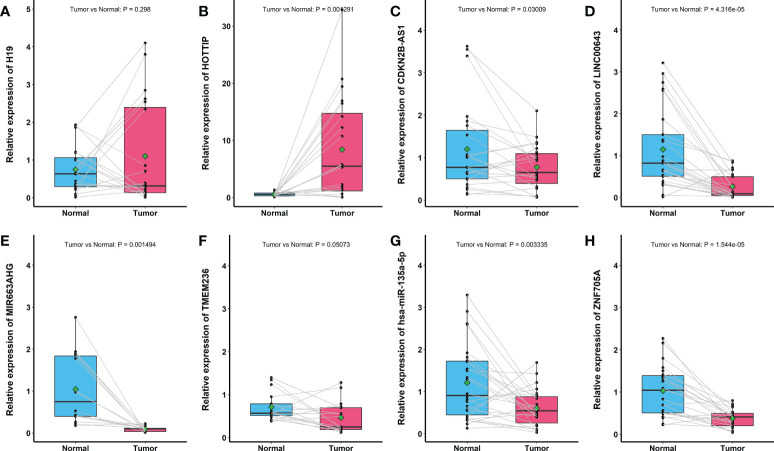
Reverse transcription–quantitative PCR result of eight RNAs expression in 30 pairs of gastric cancer tissues and adjacent non-tumor tissues. **(A)** H19. **(B)** HOTTIP. **(C)** CDKN2B-AS1. **(D)** LINC00643. **(E)** MIR663AHG. **(F)** TMEM236. **(G)** hsa-miR-135a-5p. **(H)** ZNF705A.

**Figure 13:**
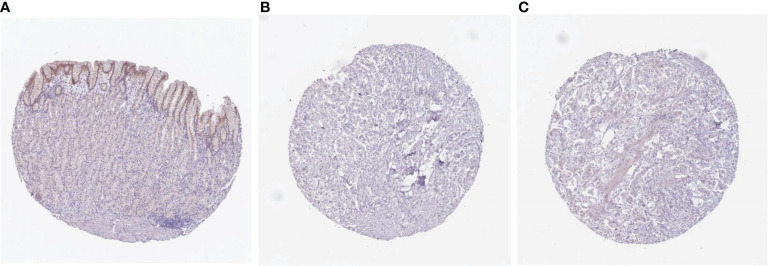
Comparison of TMEM236 expression at the protein level immunohistochemistry pictures. **(A)** normal (left) and **(B, C)** tumor (middle and right) tissues.

To further verify the expression or abundance of prognostic genes and immune cells constructing ELNs markers, meta-analysis was performed. We performed univariate Cox regression analysis for partial markers (in the absence of miRNAs in all data sets in GEO, we were unable to verify the prognostic value of hsa-miR-135a-5p) in the ELNs signature based on seven GEO data sets, with available OS data and clinical information. Then, a meta-analysis based on the univariate Cox regression analysis results of eight GC cohorts including TCGA STAD and seven GEO data sets was conducted, integrating the HR values of these markers from multiple data sets to assess their impact on prognosis (**Figures S7A–I**). The meta-analysis results exhibited that HOTTIP and LINC00643 were associated with the OS of GC. According to the fixed-effects model, HOTTIP was remarkably downregulated in the GC group (HR = 1.40, 95% CI: 1.10–1.78, Z = 2.73, p < 0.01). The combined HR of LINC00643 was 1.80, according to the random-effects model (95% CI: 1.31–2.41, Z = 3.60, p < 0.01), indicating that LINC00643 was lowly expressed in GC. Among the remaining makers, the expression of MIR663AHG, neutrophils, and ZNF705A was not significant with OS, but the tendency was consistent with the survival analysis and Cox regression of TCGA. The ELNs signature we built was based on tumor sample data analysis; to further validate the expression of the prognostic genes constructing the ELNs signature, we analyzed the difference in seven RNAs (CDKN2B-AS1, H19, HOTTIP, LINC00643, MIR663AHG, TMEM236, and ZNF705A) between the normal samples and tumor samples. We compared the lncRNAs and mRNAs expression levels in GC tissues and normal gastric tissues in the GEPIA database, and the results showed that CDKN2B-AS1, H19, HOTTIP, MIR663AHG, and ZNF705A were highly expressed in GC, whereas LINC00643 and TMEM236 exhibited low expression in GC (**Figures S8A–G**). Subsequently, we detected the expression levels of eight RNAs in GC tissues (n = 30) and adjacent non-tumorous tissues (n = 30) by an RT-qPCR assay (**Figure 12**). Consistent with our bioinformatics analysis results, the results of an RT-qPCR experiment showed that the RNA expressions of LINC00643 (p < 0.0001; **Figure 12D**), TMEM236 (**Figure 12F**, paired t-test, p = 0.05073), and hsa-miR-135a-5p (p = 0.003335; **Figure 12G**) were downregulated in GC tissues compared with adjacent non-tumorous tissues. Furthermore, to evaluate TMEM236 and ZNF705A (not found) expressions at the protein level, the IHC result provided by the HPA database was analyzed, and we compared the results of the TMEM236 gene in the HPA (protein expression level) database and the TCGA database (gene expression level). As shown in **Figures 13A–C**, the data analysis results of the two databases are consistent. Normal gastric tissues had moderate TMEM236 IHC staining, whereas tumor tissues had weak staining.

#### Construction and evaluation of a prognostic nomogram for patients with gastric cancer

**Figure 14:**
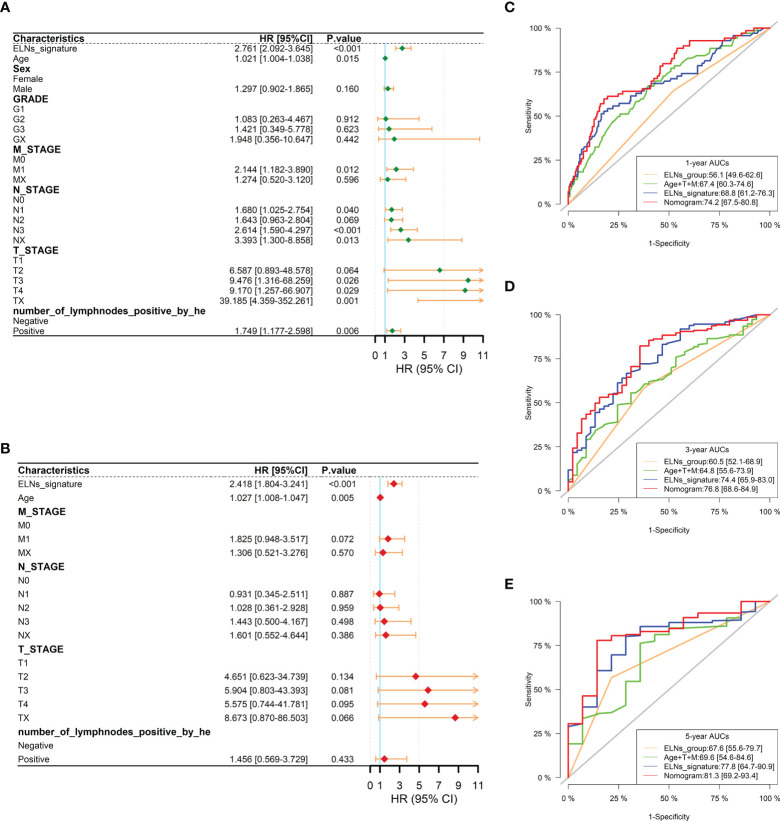
Prognostic analysis of the gastric cancer patients in the TCGA cohort. **(A, B)** Forest plots of univariate and multivariate Cox regression analysis between the ELNs signature and clinicopathological characteristics regarding OS in the TCGA cohort. **(C–E)** Time-dependent receiver operating characteristic analyses were constructed by the ELNs signature, ELNs group, age + T stage + M stage, etc., to show their prognostic ability in the TCGA cohort. ELNs, the number of lymph nodes examined; OS, overall survival.

**Figure 15:**
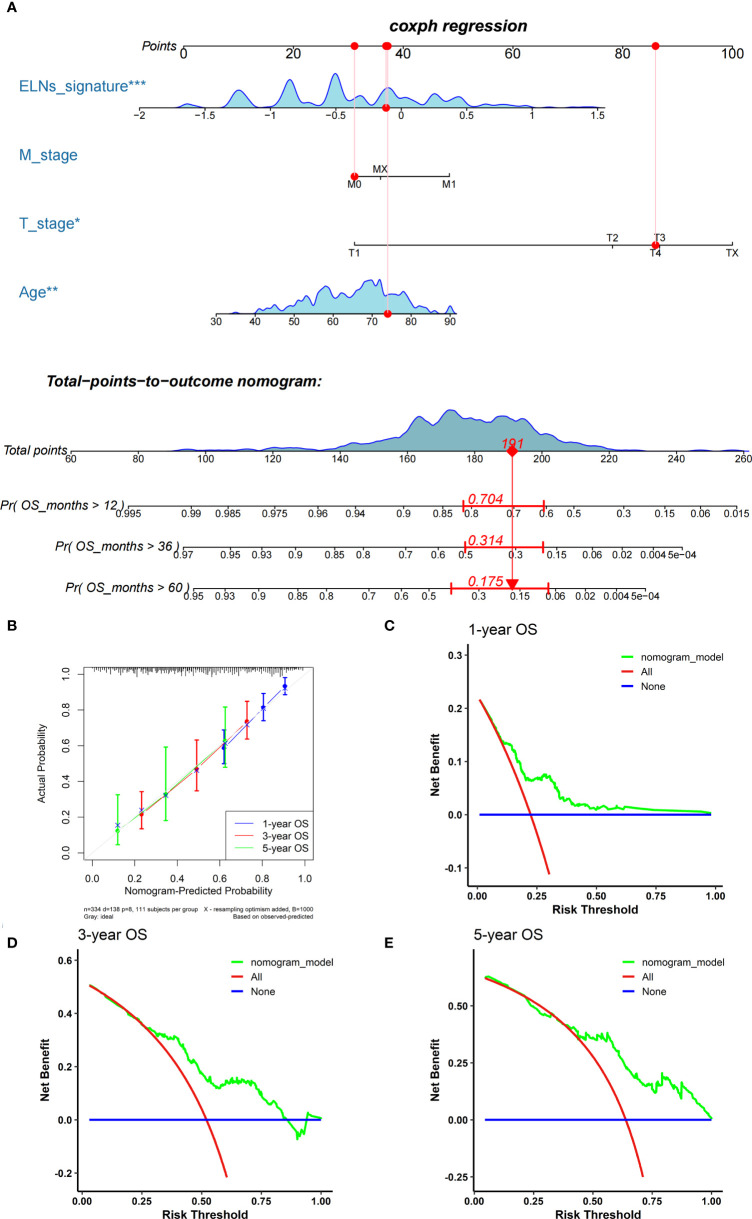
Establishment and assessment of the nomogram. **(A)** The nomogram plot was built based on the ELNs signature, age, M stage, and T stage. **(B)** The calibration curves showed that the predicted OS of the nomogram is highly concordant with the actual OS. **(C–E)** DCAs of the nomogram for 1-, 3-, and 5-year OS in the TCGA cohort. OS, overall survival; DCA, decision curve analysis. *p < 0.05; **p < 0.01; ***p < 0.001.

We carried out univariate and multivariate Cox regression analyses to study whether the ELNs signature was an independent prognostic factor for the OS of GC patients (**Figures 14A, B**). As the results demonstrated, ELNs signature (HR = 2.761, 95% CI: 2.092–3.645, p < 0.001), age, M stage, N stage, T stage, and number of lymph nodes positive were potential indicators associated with the OS of GC patients in the univariate Cox regression analysis. Subsequently, the multivariable Cox regression analysis showed that the ELNs signature derived from the 11 factors (HR= 2.418, 95% CI: 1.804–3.241, p < 0.001), age, M stage, and T stage were independent prognostic factors for OS (p < 0.1). In time-dependent ROC curves analysis, the ELNs signature also exhibited better prognostic value of 1-, 3-, and 5-year survival (AUCs = 0.688, 0.744, and 0.778) than other clinical characteristics (ELNs group, AUCs = 0.561, 0.605, and 0.676; age + T stage + M stage, AUCs = 0.674, 0.648, and 0.696). Moreover, for the 1-, 3-, and 5-year OS probability, the ROC curves also showed that the combination (AUCs [95% CI] = 0.742 [0.675–0.808], 0.768 [0.686–0.849], and 0.813 [0.692–0.934]) of the ELNs signature and other independent clinicopathological prognostic factors was better than the model built only by the ELNs signature (**Figures 14C–E**). Eventually, according to the results of the ROC analyses, all independent factors were combined to create a nomogram for predicting the 1-, 3-, and 5-year OS of GC patients. We can calculate each feature’s score for each patient to predict their 1-, 3-, and 5-year OS probability, contributing to personalized precision treatment. As shown in **Figure 15A**, the contribution of the ELNs signature to the total score is greater than that of other variables. With increasing total scores, the 1-, 3-, and 5-year OS rates of GC patients decreased. Our model’s C-index reached 0.710 (95% CI: 0.663–0.753). There was a remarkable agreement between the predicted and actual 1-, 3-, and 5-year survival probabilities (**Figure 15B**). Similarly, the DCAs constructed using the TCGA cohort showed that the nomogram performed well at predicting the 1-, 3-, and 5-year OS rates in GC patients and achieved a higher net benefit (**Figures 15C–E**).

#### Somatic mutation analysis of high– and low–the number of lymph nodes examined signature groups

**Figure S9A** shows the top 20 most frequently mutated genes in the high-ELN signature and low-ELN signature GC samples. In this study, more significant co-occurrence mutations were observed among the mutations of these genes (**Figure S9B**). Subsequently, differential mutations were detected between the two groups, and the mutation burden of SYNE1 and PCDH15 genes in the high-ELN signature group was higher than that in the low-ELN signature group (**Figure S9C**).

#### Association between the prognostic the number of lymph nodes examined signature and clinical characteristics, immune checkpoints, and drug sensitivity

We further investigated the correlations of the ELNs signature with clinical features, ICP molecules, and drug sensitivity, respectively, in this section. The results of the Kruskal–Wallis rank-sum test revealed that the ELNs signature in the NX subtype was obviously higher than those in N0, N1, N2, and N3 subtypes (all p’s < 0.05; **Figure S10E**). For the T stage, the ELNs signature of GC patients with T1 was lower than those with T3, T4, and TX (all p’s < 0.05; **Figure S10F**). However, there was no difference in the ELNs signature between men and women (p = 0.18; **Figure S10A**), and lymph nodes negative and positive (p = 0.98; **Figure S10B**). As shown in **Figures S10C–D**, the M stage (p = 0.38) and the grade level (p = 0.31) were not related to the ELNs signature. The finding of this study suggested that ELNs signature may play a pivotal role in the development of GC.

Recently, ICPs have been recognized as potential therapeutic targets for many malignant tumors and have been used in tumor immunotherapy. Therefore, to explore whether the ELNs signature could predict immunotherapeutic benefits in GC patients, we further explored the difference in the expression of ICP genes between the two groups. We extracted the expression of 50 ICPs (**Figure S11A**), namely ADORA2A, BTLA, BTNL2, CD160, CD200, CD200R1, CD244, CD27, CD274, CD276, CD28, CD40, CD40LG, CD44, CD48, CD70, CD80, CD86, CTLA4, HAVCR2, HHLA2, ICOS, COSLG, IDO1, IDO2, KIR3DL1, LAG3, LAIR1, LGALS9, NRP1, PDCD1, PDCD1LG2, TIGIT, TMIGD2, TNFRSF14, TNFRSF18, TNFRSF25, TNFRSF4, TNFRSF8, TNFRSF9, TNFSF14, TNFSF15, TNFSF18, TNFSF4, TNFSF9, VTCN1, ENTPD1, NT5E, SIGLEC15, and NCR3, to assess their relationships with the ELNs signature. As presented in **Figure S11B**, CD40 and VTCN1 expression levels in the high-ELNs signature group patients were markedly higher than those in the low-ELNs signature group patients (p < 0.05). The expression of CD44, ENTPD1, NT5E, and NRP1 in the low-ELN signature was significantly higher than that in the high-ELN signature (p < 0.05) (**Figures S11B, S12**). Meanwhile, we also evaluated the correlation between the ELNs signature and CD40, VTCN1, CD44, ENTPD1, NT5E, and NRP1 (**Figures S11C–H**). The ELNs signature was positively correlated with the expression of CD40 (p = 0.041) and VTCN1 (p = 0.001), whereas CD44 (p = 0.025), ENTPD1 (p = 0.0014), NT5E (p = 0.0053), and NRP1 (p = 0.003) were negatively correlated with the ELNs signature. This result indicated that patients in the low-ELN signature group have a better effect with immunotherapy.

Besides checkpoint blockade therapy, we compared the sensitivity of high- and low-ELN signature groups to chemotherapy drugs. Based on the GDSC database, we forecasted the chemotherapy response of high- and low-ELN signature groups. The results displayed that a total of 31 targeted agent drugs had an obvious difference in the IC_50_ between high- and low-ELN signature groups in GC (**Figure S13**). Among those outcomes, the estimated IC_50_ levels of BIRB-0796 (Doramapimod), BMS-708163 (Avagacestat), GW-441756, PF-4708671, rapamycin, and sorafenib in the high-ELN signature group were significantly lower than those in the low-ELN signature group (**Figure S13A**), and the remaining results showed that the estimated IC_50_ levels of other drugs in the high-ELN signature group were significantly higher than those in the low-ELN signature group (**Figure S13B**), which indicated that patients with low-ELN signature were more sensitive to these chemotherapeutics.

## Discussion

Lymph node metastasis plays an important role in GC long-term survival and recurrence (12). Appropriate staging of lymph node metastasis can accurately predict and improve the prognosis of patients. The ability to adequately evaluate lymph node metastasis depends on the total number of detected lymph nodes that can be used for histological evaluation. In addition, a large number of studies have demonstrated that lymph node retrieval with a sufficient number and dissection regions is necessary for proper N staging and setting up of appropriate GC treatment regimens. According to those findings, it confirmed that the number of lymph nodes was an independent predictor of the GC prognosis. Totally, a greater number of ELNs were associated with more precise nodal staging, and the incremental number of lymph node retrieval is directly correlated with improved survival (13–18). Unfortunately, because of insufficient ELNs numbers, GC can be incorrectly staged, which is called “staging migration.” Correct staging is the basis of the optimal strategy for adjuvant therapy, and patients who are underestimated may miss out on access to adjuvant therapy, resulting in adverse outcomes (15, 19). There may be a variation in the number of lymph node examinations depending on the surgical method (20). Furthermore, it is possible for the dissection procedure of the surgeon to influence not only the number of examined lymph nodes but also the pathologist’s search for lymph nodes (21, 22). Consequently, it may be necessary for the pathologist to examine the specimen with high quality to find more positive lymph nodes and provide more accurate staging (23).

To date, there is no agreement on the number of regional lymph nodes to be retrieved for adequate staging, and the optimal number of ELNs continues to be a contentious topic, but the AJCC 8th GC staging system recommends the examination of at least 16 lymph nodes. Moreover, a majority of previous studies have demonstrated a close relationship between ELNs and GC outcomes. However, not much has been done to improve prognosis accuracy using bioinformatics, clinicopathological factors, and machine learning. For example, the true impact of ELNs on OS may be understated because of the absence of a special ELNs assessment signature. From the macroscopic and molecular levels, as a result of combining two widely used external databases (SEER and TCGA), we were able to provide novel insights regarding the relationship between ELNs-related DERNAs, immune cells, and the survival of GC patients. Moreover, our study also provides an effective ELNs signature and nomogram model for evaluating the GC prognosis.

First, we developed an accurate nomogram prediction model of GC patients’ OS using the large cohort in the SEER database. Before establishing the nomogram prediction model, according to the stratified K-M curve analysis by clinicopathological features, we explored the effect of ELNs on survival. Our study results revealed that, for the N0, N1, and N2 patients, there were wide differences in survival between the high- and low-ELN groups; higher excess hazard (lower survival) was observed in patients with ≤16 ELNs than in patients with >16 ELNs. However, in the results of N3 and NX patients, the differences in survival between the two groups were not significant, regardless of the number of lymph nodes examined. Furthermore, a similar pattern of findings was observed for patients stratified as follows: grade and lymph node status. These results demonstrated that >16 ELNs were a prerequisite for the accurate evaluation of prognosis in GC patients. This study further indicated that, in our nomogram, the study of large cohorts of GC patients revealed that ELNs were independent prognostic factors, and ELNs presented to be a protective factor (high *vs*. low, HR = 0.659, 95% CI: 0.626–0.694, p < 0.0001), indicating that the survival was worse for patients whose number of lymph nodes examined was less than the optimal number (16) based on the SEER database. It was found that patients with more examined lymph nodes tended to have higher survival rates, as in previous studies (8, 18). Synthesizing above all outcomes, we can clearly know that a greater number of lymph nodes can reduce the likelihood of undetected positive lymph nodes, improving the quality of adjuvant chemotherapy and improving long-term survival. In gathering fewer lymph nodes, there is an increased chance of missing positive nodal disease, and this may lead to inappropriate patient selection and improper adjuvant therapy selection (8). Based on the optimal cutoff value of ELNs established by the SEER cohort, the TCGA and Chinese cohorts were separated into two subgroups (high and low ELNs). Meanwhile, the nomogram model was externally validated using the TCGA database and the Chinese cohort. As a result of this study, the predicted values of the model are in good agreement with the actual values for the two external validation data sets.

Then, we analyzed the DERNAs between the high- and low-ELN groups based on lncRNA, mRNA, miRNA, clinical, and immune cell fraction data from 334 GC samples collected from TCGA. A total of 664 DEmRNAs, 530 DElnRNAs, and 20 DEmiRNAs were identified. Ultimately, a GC-specific ceRNA network containing 13 mRNAs, 4 miRNAs, and 6 lncRNAs was created by integrating the interaction between DEmiRNAs and DEmRNAs or DElncRNAs. In the ceRNA network, HOTTIP had the highest connection degree within the prognostic DElncRNAs including MIR663AHG, LINC00643, HOTTIP, CDKN2B-AS1, and H19. Excepting lncRNAs, miRNAs should also get comprehensive attention. We observed that the DEmiRNA hsa-miR-135a-5p kept the highest connection degree among the prognostic DEmiRNAs in the ceRNA network. Among the prognostic DEmRNAs, APOA1 and ARC had the same connection degree in the ceRNA network. Therefore, we concluded that they might exert a strong influence on GC pathogenesis. Subsequently, by understanding the immune microenvironment, we found that the fractions of plasma cells, neutrophils, Tregs, NK cells resting, dendritic cells resting, dendritic cells activated, and mast cells resting were different between high- and low-ELN groups, suggesting that the ELNs status could change the immune microenvironment to affect prognosis. In addition, the correlation analysis showed that hsa-miR-135a-5p was associated with neutrophils and plasma cells significantly. Meanwhile, APOA1 was associated with Tregs significantly. We could infer that the three pairs and their relevant mechanisms would play essential roles in the prediction and remedy of the GC prognosis. Furthermore, to obtain the markers with the greatest potential prognostic values, univariate Cox regression analysis, LASSO regression analysis, and multivariate Cox regression analysis were performed to identify 11 OS-related markers (ELNs group, TMEM236, ZNF705A, MIR663AHG, LINC00643, HOTTIP, CDKN2B-AS1, H19, hsa-miR-135a-5p, neutrophils, and Tregs) and construct an OS-related ELNs signature. In the signature model, Tregs, H19, HOTTIP, LINC00643, TMEM236, and hsa-miR-135a-5p were unfavorable factors for GC prognosis, whereas other factors showed a protective effect on the outcome.

Our prognostic signature containing 10 biomarkers (TMEM236, ZNF705A, MIR663AHG, LINC00643, HOTTIP, CDKN2B-AS1, H19, hsa-miR-135a-5p, neutrophils, and Tregs) can identify GC patients with a high risk of poor prognosis. Certain genes and immune cells in the signature are related to the formation and regulation of tumor progression. For example, risk stratification plays a critical role in the early detection of GC, which can improve the cure rate and reduce mortality. LINC00643 as an epigenetic risk marker has been emphasized as a prospective biomarker for cancer risk stratification (24–31). TMEM236 is a new gene significantly downregulated in colorectal tumors (32). However, there are no studies on TMEM236 and its correlation with GC. TFs (Transcription Factors) that are specifically expressed in an individual tissue or cancer may be potential marker genes. ZNF705A was specifically highly expressed in germ cell tumors, which may be potential targets for cancer therapy (33). CDKN2B-AS1 has been confirmed to be upregulated in a variety of tumor tissues (34–39), which is involved in the processes of tumor cell proliferation, migration, invasion, and inhibition of tumor cell apoptosis. Deng et al. (39) found that GC patients with high expression of CDKN2B-AS1 had poor survival, and mechanism studies showed that CDKN2B-AS1 promoted tumor progression mainly by enhancing NF-κB signal. Our study is consistent with this study, indicating that CDKN2B-AS1 may serve as a potential biomarker and therapeutic target for the prognosis and treatment of GC. In numerous tumors, such as tongue squamous cell carcinoma (40), lung cancer (41), bladder cancer (42), and colorectal cancer (43), miR-135a-5p manifested pro-proliferation and pro-metastasis effects. Consistent with our result, by measuring the level of miR-135a-5p in samples of human GC, Zhang et al. (44) demonstrated that miR-135a-5p is typically reduced in GC tissues. Nevertheless, the role of this miRNA in GC and its specific mechanism need to be further investigated. In addition, MIR663AHG requires more explorations because its expression affects the OS while the associated mechanism remains unclear. H19 was upregulated in GC tissues, which induced tumor growth and metastasis through the miR−22−3p/Snail1 signaling pathway (45). Numerous previous studies have exhibited that high HOTTIP expression was relevant to larger tumor size, poor differentiation, deeper invasion depth, positive lymph node metastasis, advanced TNM stage, and poor overall patient survival (46–48). Tregs, as a subtype of CD4+ T cells, accumulate in the TME and play vital roles in tumor metastasis (49). Large populations of FOXP3+ Tregs have been recognized in the TME, and their accumulation has been linked to poor prognosis in cancer (50, 51). Elevated FOXP3+ Tregs have been linked to poor OS and tumor metastasis in GC (52, 53). Wang et al. (54) demonstrated that activated neutrophils with an immunosuppressive phenotype are greatly concentrated in GC, are associated with disease progression, and are inversely correlated with patient survival after surgery. Neutrophils contribute to the inhibition of antitumor immunity and the development of GC by suppressing T-cell activity in a PD-L1–dependent manner. This report is consistent with our present observation, as we observed that OS rates were considerably lower for individuals in the greater neutrophil number group in the TCGA-STAD cohort. However, apart from H19 and HOTTIP, the other RNAs were seldom studied in the context of a combination of transcriptional profiles and immune microenvironment. Our study identified TMEM236, ZNF705A, LINC00643, MIR663AHG, and hsa-miR-135a-5p as potential prognostic biomarkers of GC for the first time. Thus, signature genes identified in this study could provide underlying targets for experimental design in the laboratory to elucidate molecular mechanisms in GC.

In this study, meta-analysis results showed that LINC00643 was significantly associated with the OS of GC. The GEPIA database analysis found that CDKN2B-AS1, MIR663AHG, and ZNF705A were highly expressed in GC and that LINC00643 and TMEM236 have low expression in GC. Previous K-M survival analysis showed that CDKN2B-AS1, MIR663AHG, and ZNF705A overexpression is associated with a poor prognosis, whereas LINC00643 and TMEM236 overexpression is associated with a good prognosis. In addition, by studying GC pathological specimens, we confirmed that LINC00643, TMEM236, and hsa-miR-135a-5p were lowly expressed in GC tissues. Furthermore, TMEM236 mRNA expression showed the same results as the HPA database. These results are consistent with our Cox regression analysis in the TCGA cohort, which suggests that they may play an important role in tumorigenesis. There are few reports on the expression pattern and function of these genes in GC; we need a large number of cohort and basic experiments to further explore the potential mechanism of these genes in the future.

There have been reports on lncRNA signatures and miRNA signatures for GC. A previous study reported a three-miRNA signature that can predict outcomes in patients with GC (55). Guo et al. (56) constructed a four-lncRNA signature and successfully used a publicly available data set (GSE62254) to corroborate the reliability of the four lncRNA signatures. Recent studies have been performed to investigate the immune characteristics of GC patients, which have adequately demonstrated high prognostic potential and clinical guidance values relative to the conventional clinical characteristics or risk models (57–60). These studies have assessed the immunological characteristics of GC mainly from the perspective of immune cell infiltration. The biomarkers included in the risk model constructed by the above studies are relatively single, and the risk model established by combining multiple types of indicators may have better performance in prediction. In this study, we concentrated our efforts on exploring the immune infiltrating cell and gene associated with ELNs and established an ELNs signature associated with prognosis. According to the ROC analysis of the TCGA data sets, the ELNs signature is good at predicting short-term (1 and 3 years) and long-term (5 years) survival for GC patients. In addition, risk stratification by the ELNs signature showed that patients in the high-ELN signature subgroup had a shorter OS than those in the low-ELN signature subgroup. A nomogram integrating the ELNs signature and other clinical variables (age, M stage, and T stage) was created to provide clinicians with a quantitative approach to predict the prognosis for GC patients, which provided more precise short- and long-term survival predictions than any individual prognostic factor for GC patients. C-indices, calibration plots, and DCAs also demonstrated the excellent predictive performance of the nomogram. According to these findings, the ELNs signature was an effective predictor of the prognosis of GC patients, which has certain implications for clinical treatment decisions.

Furthermore, a comprehensive analysis of the correlation between clinicopathological characteristics, ICPs, drug sensitivity, and the prognostic signature was done. There were no significant differences in the expression of common checkpoint genes such as PD-1, PD-L1, and CTLA-4 between the two risk groups, which signified that our risk model could not predict the therapeutic effect of existing PD-1/PD-L1 or even CTLA-4 immune therapy. At present, novel immunotherapies like anti–PD-1 and anti–PD-L1 have been applied in GC. Nevertheless, only a minority of subjects benefit from immunotherapies (61, 62). However, we found that the expressions of some novel checkpoint genes (CD44, ENTPD1, NT5E, and NRP1) were upregulated in the low-ELN signature group, and our study has uncovered that the high expression of NT5E was obviously associated with poor prognosis, cancer cell migration, and metastasis in GC patients (63), and this may be served as a therapeutic target for GC metastasis. Meanwhile, the data also indicated that this signature was closely associated with immunotherapy, and the low-ELN signature patients may have a better response to immunotherapy. This is in light of the fact that most GC patients are at an advanced stage of the disease, which makes prevention and treatment of GC a high priority (64). The localized GC can only be cured by radical surgery with or without chemotherapy beforehand. However, chemotherapy is the predominant treatment method for metastatic GC (65). Unfortunately, chemotherapy shows relatively little response because of tumor heterogeneity (66). According to the estimated IC_50_, our data indicated that patients with low ELNs were more sensitive to those drugs than those with high ELNs. Patients in the high-ELN signature subgroup showed sensitive chemotherapy response only to BIRB-0796 (doramapimod), BMS-708163 (avagacestat), GW-441756, PF-4708671, rapamycin, and sorafenib. Based on the patient’s TME, medical staff can choose a suitable treatment method for the patient more accurately. ICP inhibitor therapy has recently been transformed from a single therapeutic medication pattern to a combination therapy design. The approach of combining immunotherapy with chemotherapy has been studied in a number of clinical studies. Studies about GC have shown that, compared with chemotherapy alone, combination therapy can increase the efficacy of cancer treatment. However, because chemotherapy has negative side effects, discovering the most optimal combination of chemotherapy and ICIs is critical for adopting more effective clinical strategies for treating GC patients (67, 68).

Taking our research in its entirety, there are some obvious strengths. It is clear from this study that the GC cohorts from the multicenter study had large sample sizes. Utilizing the public SEER, TCGA, and GEO databases and an external cohort from the Affiliated Tumor Hospital of Xinjiang Medical University, we identified and comprehensively analyzed the ELNs associated with the prognosis of GC patients. More importantly, our research is the first to use a large clinical database and a large-scale omics database to establish a signature related to ELNs for predicting the prognosis of GC patients. The ELNs signature was developed to predict outcomes for patients, showing satisfactory prediction performance. With the ELNs signature and other significant clinical indicators, a novel nomogram was able to comprehensively and systematically demonstrate the predicted effects. Second, even people without medical backgrounds can perform the calculations, making it possible to apply the signature to a variety of different settings.

Still, there are some limitations in this study. First, since the study employed a retrospective research design, some critical information about the patients might have been omitted inevitably, reducing the number of eligible participants. Second, we have internally verified the nomogram prediction model based on ELNs signature, and the findings of this study would be more meaningful if this model could be well validated externally with another real-world, independent, large-quantity, high-quality cohort, and thus, a more diverse patient population could be extrapolated. However, the application of the prognostic prediction model based on the ELNs signature required four types of data, containing clinical information, RNA-seq, miRNA-seq, and an abundance of TIICs, which involve high costs and are not easily feasible in practice. There are still many limitations in our model, but the findings show that it remains an instructive and efficient way for predicting the accurate individual clinical outcomes of GC patients. However, it is necessary to explore and prove further the potential value of these results in the prognosis and treatment of GC.

## Conclusion

In conclusion, our study explored the prognostic role of ELNs in GC and successfully developed an ELNs signature correlated with the GC prognosis. The results exhibited that this signature is an effective predictor of GC patients. Moreover, to predict the 1-, 3-, and 5-year OS of patients with GC, we established a novel and robust nomogram integrating the ELNs signature and clinical factors, which will help personalize survival prediction and clinical decision-making in GC patients.

## Data availability statement

The original contributions presented in the study are included in the article/**Supplementary Material**. Further inquiries can be directed to the corresponding authors.

## Ethics statement

This study was reviewed and approved by the institutional ethical review board at The Affiliated Tumor Hospital of Xinjiang Medical University. Written informed consent to participate in this study was provided by the participants’ legal guardian/next of kin.

## Author contributions

HuL, KW and DDL conceived and designed the study. HuL and DDL analyzed and interpreted the data and drafted the manuscript. HuiL provided technical support. LL, ZY and TH prepared samples and conducted experiments. KW and SZ reviewed and revised the paper. All authors contributed to the article and approved the submitted version.

## Funding

This work was supported by grants from the Tianshan Innovative Research Team of Xinjiang Uygur Autonomous Region, China (grant number 2020D14020), and the Natural Science Foundation of China (grant number 11961071).

## Acknowledgments

We sincerely thank the support of the ‘14th Five-Year’ college pharmacy specialty discipline of Xinjiang Uygur Autonomous Region. We also gratefully acknowledge the data platforms, including SEER, TCGA, and GEO databases, for making these data sets publicly available to promote continuous research.

## Conflict of interest

The authors declare that the research was conducted in the absence of any commercial or financial relationships that could be construed as a potential conflict of interest.

## Publisher’s note

All claims expressed in this article are solely those of the authors and do not necessarily represent those of their affiliated organizations, or those of the publisher, the editors and the reviewers. Any product that may be evaluated in this article, or claim that may be made by its manufacturer, is not guaranteed or endorsed by the publisher.
