# Supplementary material for: A nomogram model based on the number of examined lymph nodes–related signature to predict prognosis and guide clinical therapy in gastric cancer
Source: Front Immunol. 2022 Nov 2;13:947802. doi: 10.3389/fimmu.2022.947802 (PMC9667298; doi:10.3389/fimmu.2022.947802)
Supplement: Supplementary file 1 [file DataSheet_1.zip › data sheet/TableS.docx]

**TABLES**

**TABLE S2 |** Sequences of primers used for reverse transcription quantitative PCR

| **Gene** | **Primer sequence** |
| --- | --- |
| TMEM236 | F:TGTTAGCAAGCAGACCGCACAG  R:TACGCAGTTCACAATAGGGTTTGGG |
| miR-135a-5p | F:CGCGTATGGCTTTTTATTCCT  R:AGTGCAGGGTCCGAGGTATT |
| ZNF705A | F:TCCTCACAGCTCACCTCCCT  R:GGGCTGAAGATCCCTGGTGT |
| CDKN2B-AS1 | F:CGGACTAGGACTATTTGCCACGAC  R:CCAGGACGGAGATCAGATGAGAGG |
| H19 | F:TGATGACGGGTGGAGGGGCTA  R:TGATGTCGCCCTGTCTGCACG |
| HOTTIP | F:CCTAAAGCCACGCTTCTTTG  R:TGCAGGCTGGAGATCCTACT |
| MIR663AHG | F:GGTACACTGGTCGACAAAACC  R:TGGAGACGCTATCTCGGGTA |
| LINC00643 | F:GGCTGGTTCTTTCTGAAGTGCTTTG  R:TCTTTGCCCCATCTCGAATTGCTAC |
| GAPDH | F:GCACCGTCAAGGCTGAGAAC  R:TGGTGAAGACGCCAGTGGA |
| U6 | F:GCTTCGGCAGCACATATACTAAAAT  R:CGCTTCACGAATTTGCGTGTCAT |

**TABLE S3 |** Demographic and clinicopathological characteristics of GC patients from the different cohorts

| **Characteristics** | **Modeling cohort (SEER)** | | | | **TCGA-STAD cohort (external validation cohort)**  **(n =** **396)** | **Chinese-STAD cohort (external validation cohort)**  **(n = 471)** |
| --- | --- | --- | --- | --- | --- | --- |
|  | **Total**  **(N = 19317)** | **Training cohort**  **(n = 12880)** | **internal validation**  **cohort**  **(n = 6437)** | ***P*-value** |  |  |
| **Sex, No. (%)** |  |  |  | 0.715 |  |  |
| Male | 12157 (62.9) | 8118 (63.0) | 4039 (62.7) |  | 261 (65.9) | 351 (74.5) |
| Female | 7160 (37.1) | 4762 (37.0) | 2398 (37.3) |  | 135 (34.1) | 120 (25.5) |
| **Age, Mean (SD)** | 65.69 (13.1) | 65.67 (13.15) | 65.74 (13.0) | 0.717 | 65.33 (10.7) | 59.17 (11.03) |
| **Race, No. (%)** |  |  |  | 0.782 |  |  |
| White | 12605 (65.3) | 8425 (65.4) | 4180 (64.9) |  | 248 (62.6) | NA |
| Black | 2496 (12.9) | 1644 (12.8) | 852 (13.2) |  | 13 (3.3) | NA |
| Other | 4149 (21.5) | 2768 (21.5) | 1381 (21.5) |  | 84 (21.2) | NA |
| Unknown | 67 (0.3) | 43 (0.3) | 24 (0.4) |  | 51 (12.9) | NA |
| **Grade, No. (%)** |  |  |  | 0.104 |  |  |
| Ⅰ | 934 (4.8) | 628 (4.9) | 306 (4.8) |  | 10 (2.5) | 33 (7.0) |
| Ⅱ | 5098 (26.4) | 3370 (26.2) | 1728 (26.8) |  | 141 (35.6) | 251 (53.3) |
| Ⅲ | 12032 (62.3) | 8012 (62.2) | 4020 (62.5) |  | 236 (59.6) | 187 (39.7) |
| Ⅳ | 485 (2.5) | 324 (2.5) | 161 (2.5) |  | - | - |
| X | 768 (4.0) | 546 (4.2) | 222 (3.4) |  | 9 (2.3) | - |
| **AJCC stage, No. (%)** |  |  |  | 0.42 |  |  |
| Ⅰ | 6593 (34.1) | 4383 (34.0) | 2210 (34.3) |  | 51 (13.2) | 121 (25.7) |
| Ⅱ | 4495 (23.3) | 3042 (23.6) | 1453 (22.6) |  | 125 (32.3) | 158 (33.5) |
| Ⅲ | 4361 (22.6) | 2921 (22.7) | 1440 (22.4) |  | 172 (44.4) | 192 (40.8) |
| Ⅳ | 3631 (18.8) | 2377 (18.5) | 1254 (19.5) |  | 39 (10.1) | - |
| **T stage, No. (%)** |  |  |  | 0.612 |  |  |
| T1 | 3985 (20.6) | 2667 (20.7) | 1318 (20.5) |  | 20 (5.1) | 79 (16.8) |
| T2 | 8762 (45.4) | 5800 (45.0) | 2962 (46.0) |  | 85 (21.5) | 68 (14.4) |
| T3 | 4683 (24.2) | 3154 (24.5) | 1529 (23.8) |  | 186 (47.0) | 213 (45.2) |
| T4 | 1753 (9.1) | 1174 (9.1) | 579 (9.0) |  | 100 (25.3) | 111 (23.6) |
| TX | 134 (0.7) | 85 (0.7) | 49 (0.8) |  | 5 (1.3) | - |
| **N stage, No. (%)** |  |  |  | 0.766 |  |  |
| N0 | 7172 (37.1) | 4788 (37.2) | 2384 (37.0) |  | 117 (29.5) | 200 (42.5) |
| N1 | 7399 (38.3) | 4942 (38.4) | 2457 (38.2) |  | 112 (28.3) | 82 (17.4) |
| N2 | 3254 (16.8) | 2178 (16.9) | 1076 (16.7) |  | 78 (19.7) | 76 (16.1) |
| N3 | 1471 (7.6) | 959 (7.4) | 512 (8.0) |  | 80 (20.2) | 113 (24.0) |
| NX | 21 (0.1) | 13 (0.1) | 8 (0.1) |  | 9 (2.3) | - |
| **M stage, No. (%)** |  |  |  | 0.601 |  |  |
| M0 | 17186 (89.0) | 11478 (89.1) | 5708 (88.7) |  | 357 (90.2) | 471 (100.0) |
| M1 | 1957 (10.1) | 1290 (10.0) | 667 (10.4) |  | 24 (6.1) | - |
| MX | 174 (0.9) | 112 (0.9) | 62 (1.0) |  | 15 (3.8) | - |
| **Regional** **lymph nodes examined count, Mean (SD)** | 17.68 (12.6) | 17.69 (12.6) | 17.65 (12.5) | 0.816 | 22.07 (19.3) | 24.61 (11.0) |
| **LN** **status, No. (%)** |  |  |  | 0.984 |  |  |
| Negative | 7820 (40.5) | 5213 (40.5) | 2607 (40.5) |  | 131 (33.1) | 197 (41.8) |
| Positive | 11497 (59.5) | 7667 (59.5) | 3830 (59.5) |  | 265 (66.9) | 274 (58.2) |
| **Tumor size, Mean (SD)** | 49.78 (43.4) | 49.66 (44.0) | 50.03 (42.3) | 0.579 | NA | NA |

**Data are n, N or No. (%) unless indicated otherwise**

**TABLE S4 |** Univariate and multivariate Cox regression analyses in the training cohort

| **Characteristics** | **Univariate analysis** | | **Multivariate analysis** | |
| --- | --- | --- | --- | --- |
|  | **HR (95%CI)** | ***P* -value** | **HR (95%CI)** | ***P* -value** |
| **Sex, No. (%)** |  |  |  |  |
| Male | 1 |  | - |  |
| Female | 0.993 (0.947-1.041) | 0.763 | - | - |
| **Age, Mean (SD)** | 1.016 (1.014-1.018) | **<0.0001** | 1.023 (1.022-1.026） | **<0.0001** |
| **Grade, No. (%)** |  |  |  |  |
| Ⅰ | 1 |  | 1 |  |
| Ⅱ | 1.451 (1.272-1.655) | **<0.0001** | 1.099 (0.963-1.256) | 0.161 |
| Ⅲ | 2.004 (1.766-2.273) | **<0.0001** | 1.264 (1.112-1.438) | **<0.001** |
| Ⅳ | 2.104 (1.746-2.534) | **<0.0001** | 1.289 (1.068-1.556) | **0.008** |
| X | 1.569 (1.323-1.859) | **<0.0001** | 1.237 (1.042-1.469) | **0.015** |
| **T stage, No. (%)** |  |  |  |  |
| T1 | 1 |  | 1 |  |
| T2 | 2.385 (2.213-2.570) | **<0.0001** | 1.797 (1.660-1.945) | **<0.0001** |
| T3 | 3.746 (3.460-4.056) | **<0.0001** | 2.365 (2.166-2.582) | **<0.0001** |
| T4 | 5.035 (4.575-5.542) | **<0.0001** | 2.937 (2.646-3.260) | **<0.0001** |
| TX | 3.185 (2.292-4.428) | **<0.0001** | 2.440 (1.751-3.400) | **<0.0001** |
| **N stage, No. (%)** |  |  |  |  |
| N0 | 1 |  | 1 |  |
| N1 | 2.074 (1.959-2.196) | **<0.0001** | 0.880 (0.745-1.039) | 0.132 |
| N2 | 3.239 (3.030-3.462) | **<0.0001** | 1.341 (1.125-1.600) | **0.001** |
| N3 | 4.621 (4.236-5.040) | **<0.0001** | 2.021 (1.672-2.443) | **<0.0001** |
| NX | 1.934 (0.921-4.065) | 0.0813 | 0.927 (0.438-1.961) | 0.842 |
| **M stage, No. (%)** |  |  |  |  |
| M0 | 1 |  | 1 |  |
| M1 | 2.999 (2.809-3.202) | **<0.0001** | 1.932 (1.803-2.069) | **<0.0001** |
| MX | 1.680 (1.356-2.081) | **<0.0001** | 1.383 (1.116-1.714) | **0.003** |
| **ELNs group** |  |  |  |  |
| low | 1 |  | 1 |  |
| high | 0.862 (0.823-0.903) | **<0.0001** | 0.659 (0.626-0.694) | **<0.0001** |
| **LN status, No. (%)** |  |  |  |  |
| Negative | 1 |  | 1 |  |
| Positive | 2.594 (2.464-2.730) | **<0.0001** | 1.965 (1.672-2.310) | **<0.0001** |
| **Tumor size, Mean (SD)** | 1.0029 (1.0027-1.0032) | **<0.0001** | - | - |

**TABLE S5 |** Hypergeometric testing and correlation analysis results of ceRNAs network

| **LncRNA** | **Protein-coding RNA** | **MiRNA** | **Correlation P** | **Hypergeometric test *P*** |
| --- | --- | --- | --- | --- |
| HTR5A-AS1 | APOA1 | hsa-miR-135a-5p | 0.358702843 | 0.0625 |
| HTR5A-AS1 | ARC | hsa-miR-135a-5p | 0.100284125 | 0.0625 |
| HTR5A-AS1 | GAGE1 | hsa-miR-135a-5p | 9.57E-11 | 0.0625 |
| MIR663AHG | APOA1 | hsa-miR-135a-5p | 5.03E-05 | 0.0625 |
| MIR663AHG | ARC | hsa-miR-135a-5p | 0.86995925 | 0.0625 |
| MIR663AHG | GAGE1 | hsa-miR-135a-5p | 1.50E-13 | 0.0625 |
| LINC00643 | APOA1 | hsa-miR-135a-5p | 0.007761757 | 0.1250 |
| LINC00643 | ARC | hsa-miR-135a-5p | 0.189361832 | 0.1250 |
| LINC00643 | GAGE1 | hsa-miR-135a-5p | 0.001859995 | 0.1250 |
| HOTTIP | IL11 | hsa-miR-211-5p | 0.010088348 | 0.0625 |
| HOTTIP | GLP1R | hsa-miR-211-5p | 0.015896638 | 0.0625 |
| HOTTIP | ASGR1 | hsa-miR-211-5p | 0.092686274 | 0.0625 |
| HOTTIP | TMEM236 | hsa-miR-211-5p | 0.802914803 | 0.0625 |
| HOTTIP | SERPINF2 | hsa-miR-211-5p | 3.49E-05 | 0.0625 |
| HOTTIP | ZNF705A | hsa-miR-211-5p | 0.078816274 | 0.0625 |
| HOTTIP | SMOC1 | hsa-miR-211-5p | 0.183329906 | 0.0625 |
| CDKN2B-AS1 | OLAH | hsa-miR-512-3p | 0.011810536 | 0.1875 |
| CDKN2B-AS1 | DRAXIN | hsa-miR-512-3p | 0.045367497 | 0.1875 |
| H19 | IGDCC3 | hsa-miR-518c-5p | 2.37E-12 | 0.3500 |

***ceRNAs, competing endogenous RNAs; LncRNA, long non-coding RNA; Protein-coding RNA, mRNA; MiRNA, microRNA.**

**TABLE S7 |** Identification of the 11 factors involved in the establishment of the prognostic ELNs signature based on multivariate Cox regression analysis

| **Names** | **Coefficient** | **HR** | **95%CI** | ***P*-value** |
| --- | --- | --- | --- | --- |
| ELNs group (high vs. low) | -0.35569 | 0.701 | 0.484-1.014 | 0.060 |
| T regulatory cells (Tregs) (low vs. high) | 0.38558 | 1.470 | 1.030-2.099 | 0.034 |
| Neutrophils (low vs. high) | -0.52900 | 0.589 | 0.355-0.978 | 0.041 |
| CDKN2B-AS1 (low vs. high) | -0.36941 | 0.691 | 0.480-0.995 | 0.047 |
| H19 (low vs. high) | 0.55315 | 1.739 | 1.126-2.686 | 0.013 |
| HOTTIP (low vs. high) | 0.41669 | 1.517 | 1.072-2.148 | 0.019 |
| LINC00643 (low vs. high) | 0.36619 | 1.442 | 0.978-2.127 | 0.065 |
| MIR663AHG (low vs. high) | -0.40853 | 0.665 | 0.449-0.985 | 0.042 |
| TMEM236 (low vs. high) | 0.49878 | 1.647 | 0.982-2.761 | 0.059 |
| ZNF705A (low vs. high) | -0.32665 | 0.721 | 0.504-1.031 | 0.073 |
| hsa-miR-135a-5p (low vs. high) | 0.35357 | 1.424 | 0.950-2.135 | 0.087 |

***HR: hazard ratio, CI:confidence interval**
