# Supplementary material for: A nomogram model based on the number of examined lymph nodes–related signature to predict prognosis and guide clinical therapy in gastric cancer
Source: Front Immunol. 2022 Nov 2;13:947802. doi: 10.3389/fimmu.2022.947802 (PMC9667298; doi:10.3389/fimmu.2022.947802)
Supplement: Supplementary file 2 [file DataSheet_2.zip › Data Sheet 2/Supplementary Image/FigureS.docx]

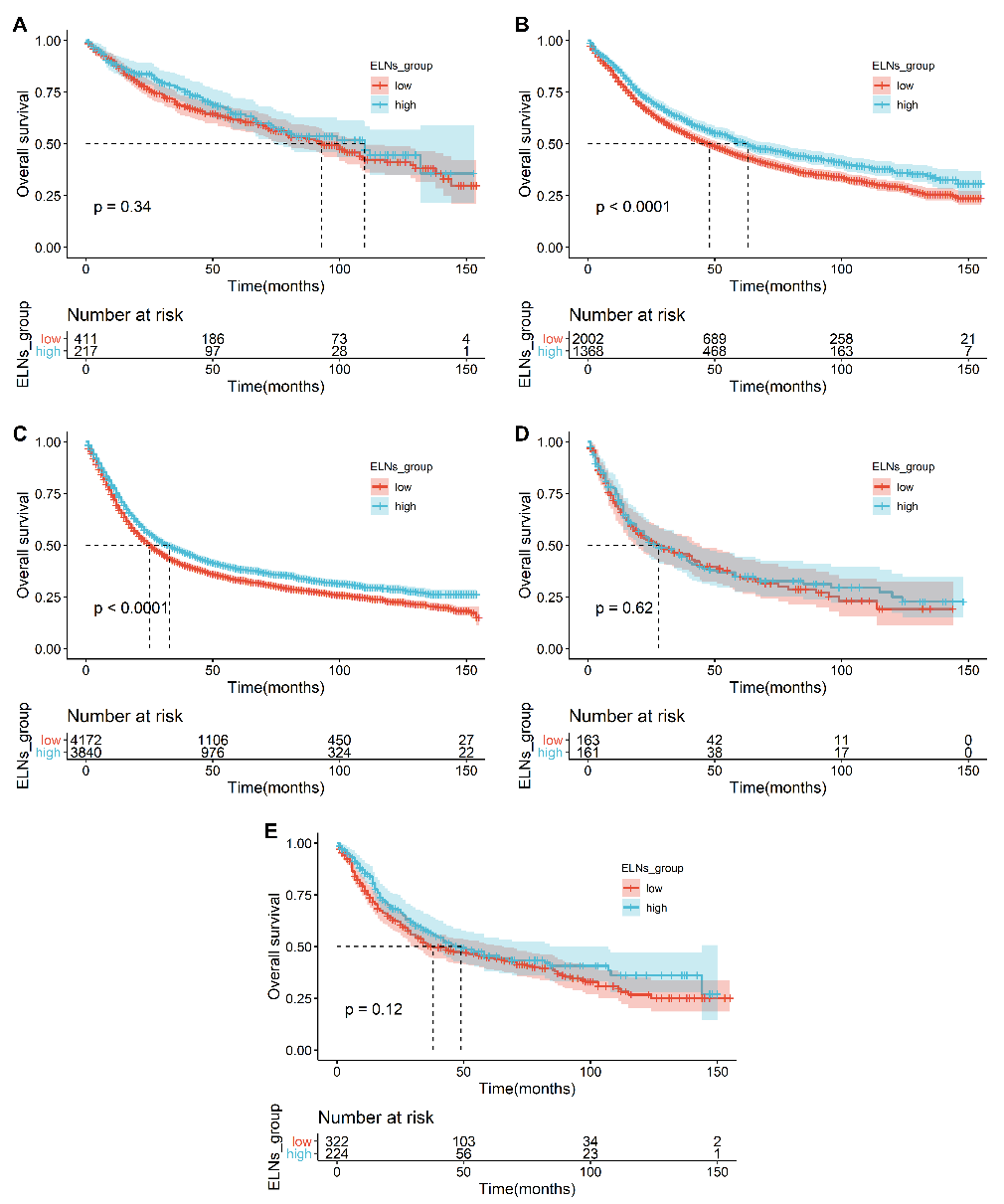


**FIGURE S1 |** Survival analyses stratified by histological grade in the training cohort. **(A)** Survival curves for OS in grade I. **(B)** survival curves for OS in grade II. **(C)** survival curves for OS in grade III. **(D)** survival curves for OS in grade IV. **(E)** survival curves for OS in grade X; ELNs, the number of examined lymph nodes; OS, overall survival.


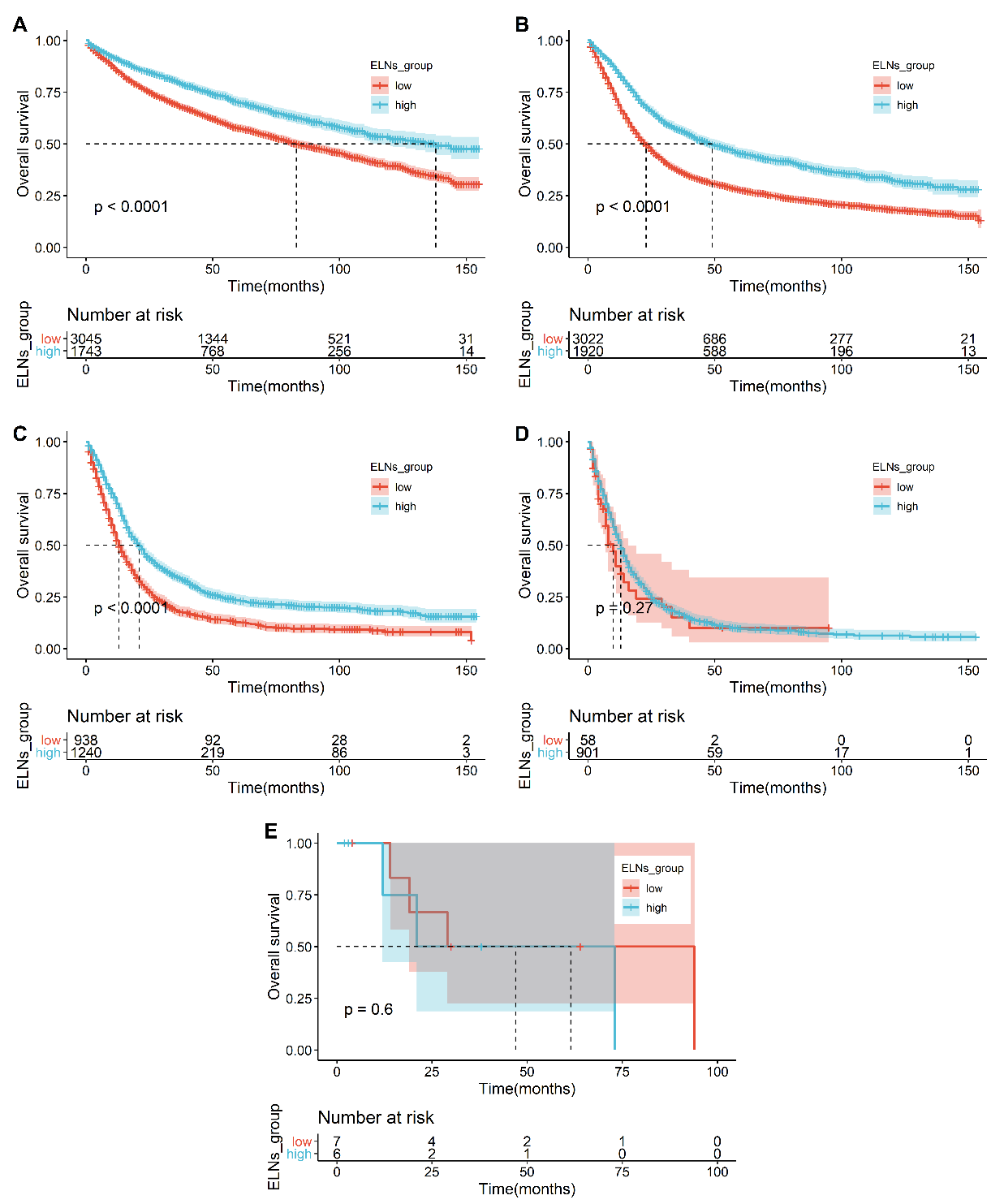


**FIGURE S2 |** Survival analyses stratified by the N stage in the training cohort. **(A)** Survival curves for OS in stage N0. **(B)** survival curves for OS in stage N1. **(C)** survival curves for OS in stage N2. **(D)** survival curves for OS in stage N3. **(E)** survival curves for OS in stage NX. ELNs, the number of examined lymph nodes; OS, overall survival.


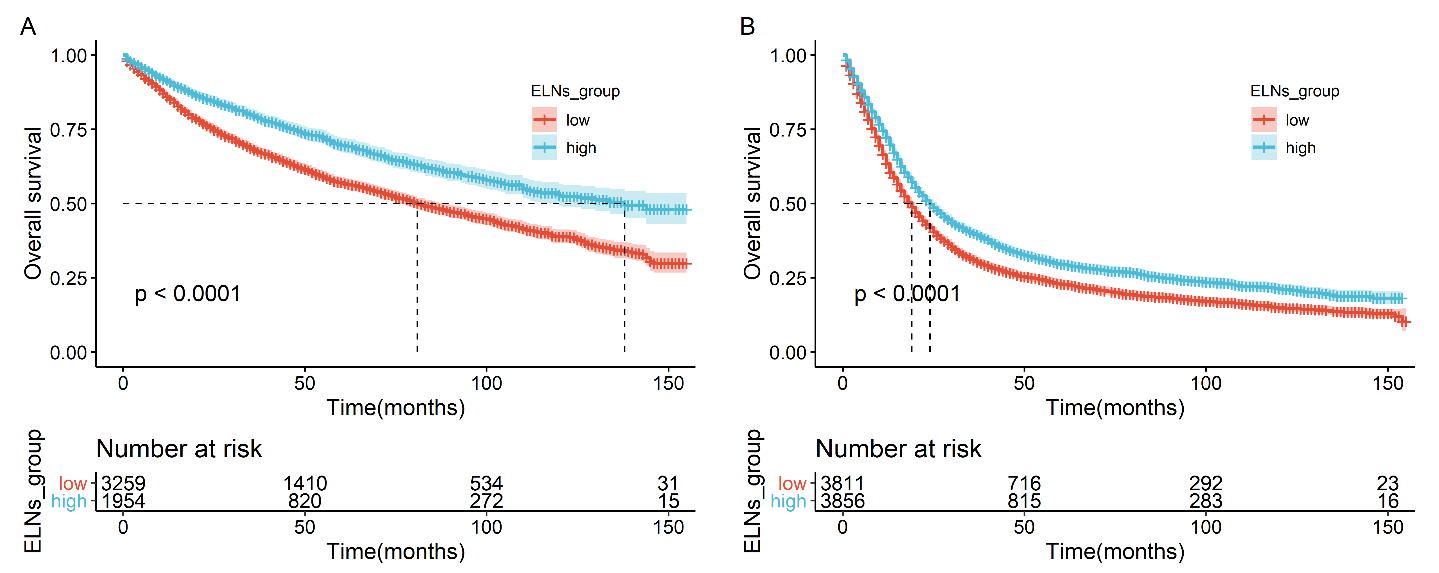


**FIGURE S3 |** Subgroup analyses of ELNs stratified by lymph node status. **(A)** OS stratified for negative lymph node group. **(B)** OS stratified for positive lymph node group. ELNs, the number of examined lymph nodes; OS, overall survival.

**
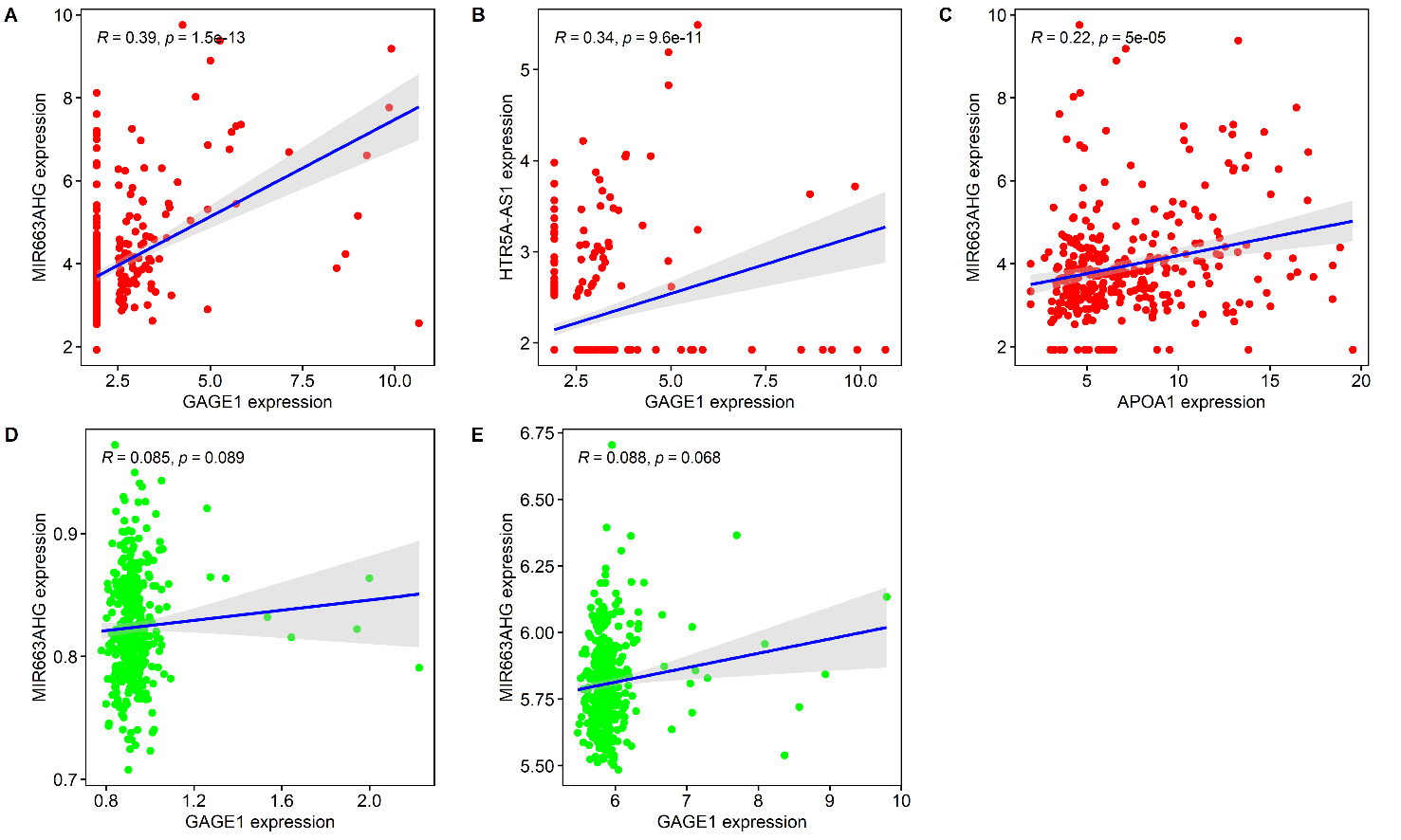
**

**FIGURE S4 |** Correlation analysis of linear regression analysis between DElncRNAs and DEmRNAs. **(A-C)** GAGE1 versus MIR663AHG, GAGE1 versus HTR5A-AS1, and APOA1 versus MIR663AHG in the TCGA cohort. **(D, E)** GAGE1 versus MIR663AHG in the GEO datasets (GSE62254, GSE84437). The gray area around the blue line represents 95% confidence interval.


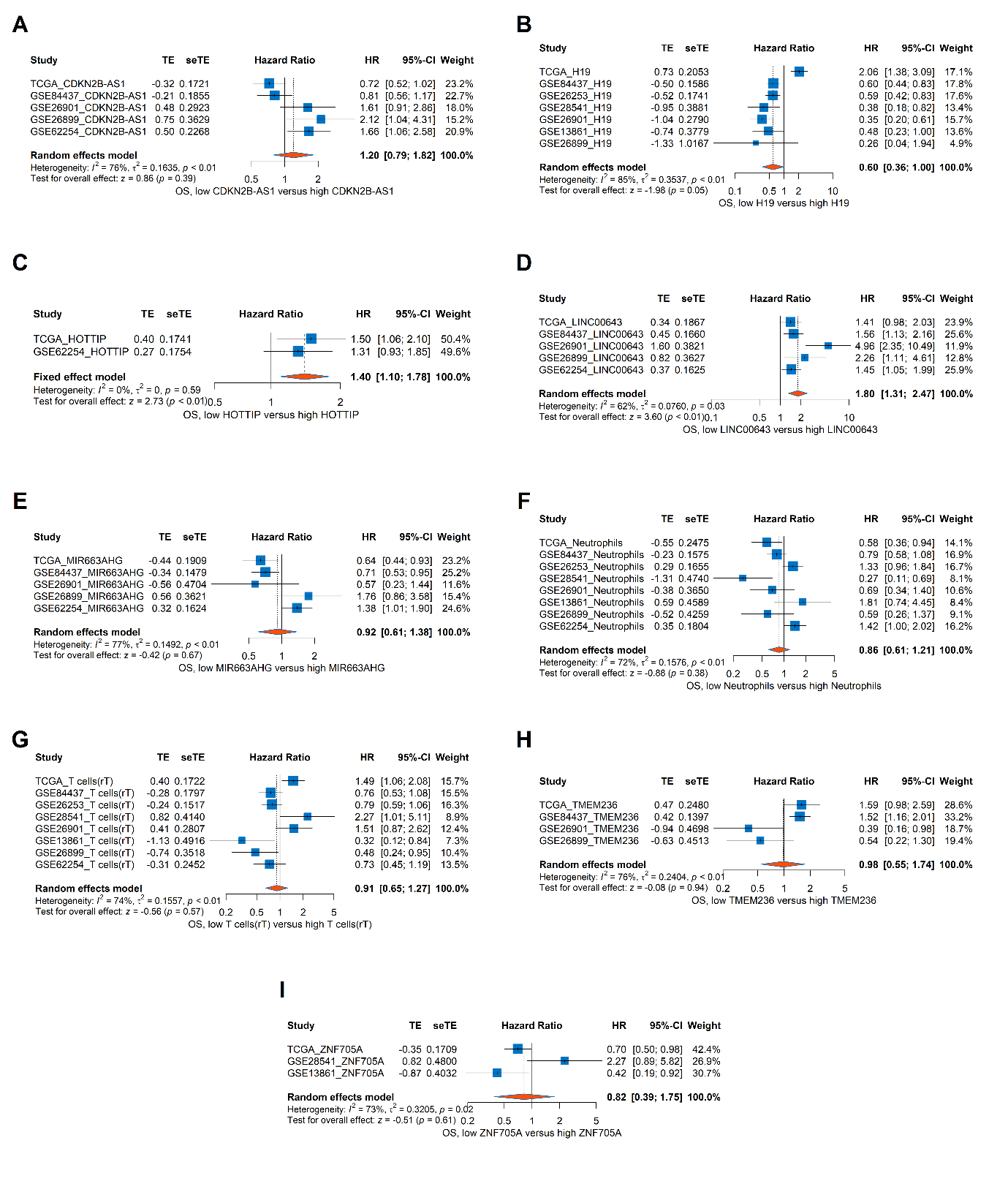


**FIGURE S7 |** A comprehensive Meta-analysis of the expression or abundance of genes and cells from the collected microarrays. **(A)** The forest plot for the pooled HR of 1.20 (95% CI: 0.79, 1.82) of CDKN2B-AS1. **(B)** The forest plot for the pooled HR of 0.60 (95% CI: 0.36, 1.00) of H19. **(C)** The forest plot for the pooled HR of 1.40 (95% CI: 1.10, 1.78) of HOTTIP. **(D)** The forest plot for the pooled HR of 1.63 (95% CI: 1.36, 1.95) of LINC00643. **(E)** The forest plot for the pooled HR of 0.92 (95% CI: 0.61, 1.38) of MIR663AHG. **(F)** The forest plot for the pooled HR of 0.86 (95% CI: 0.61, 1.21) of Neutrophils. **(G)** The forest plot for the pooled HR of 0.91(95% CI: 0.65, 1.27) of T regulatory cells (Tregs). **(H)** The forest plot for the pooled HR of 0.98 (95% CI: 0.55, 1.74) of TMEM236. **(I)** The forest plot for the pooled HR of 0.82 (95% CI: 0.39, 1.75) of ZNF705A.


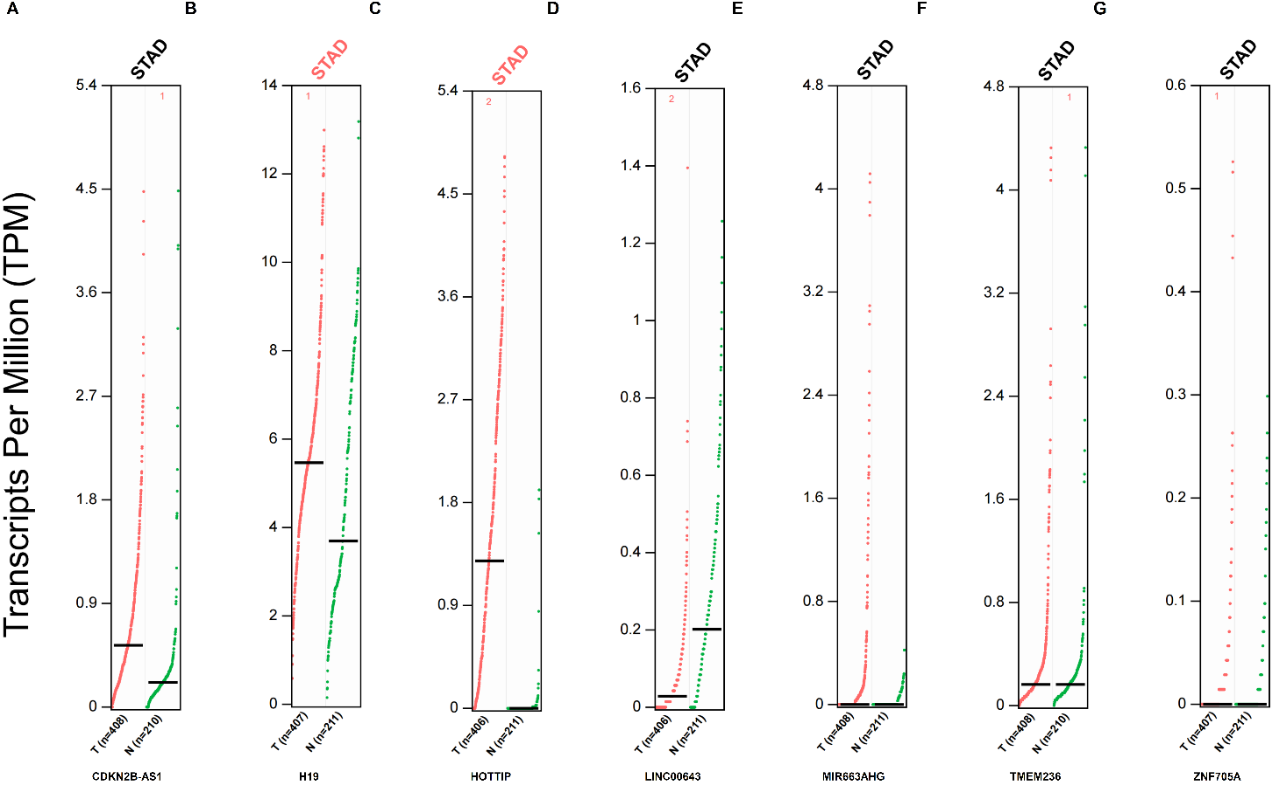


**FIGURE S8 |** A comparison of eight genes expressed in GC tissues and normal tissues from the GEPIA database.


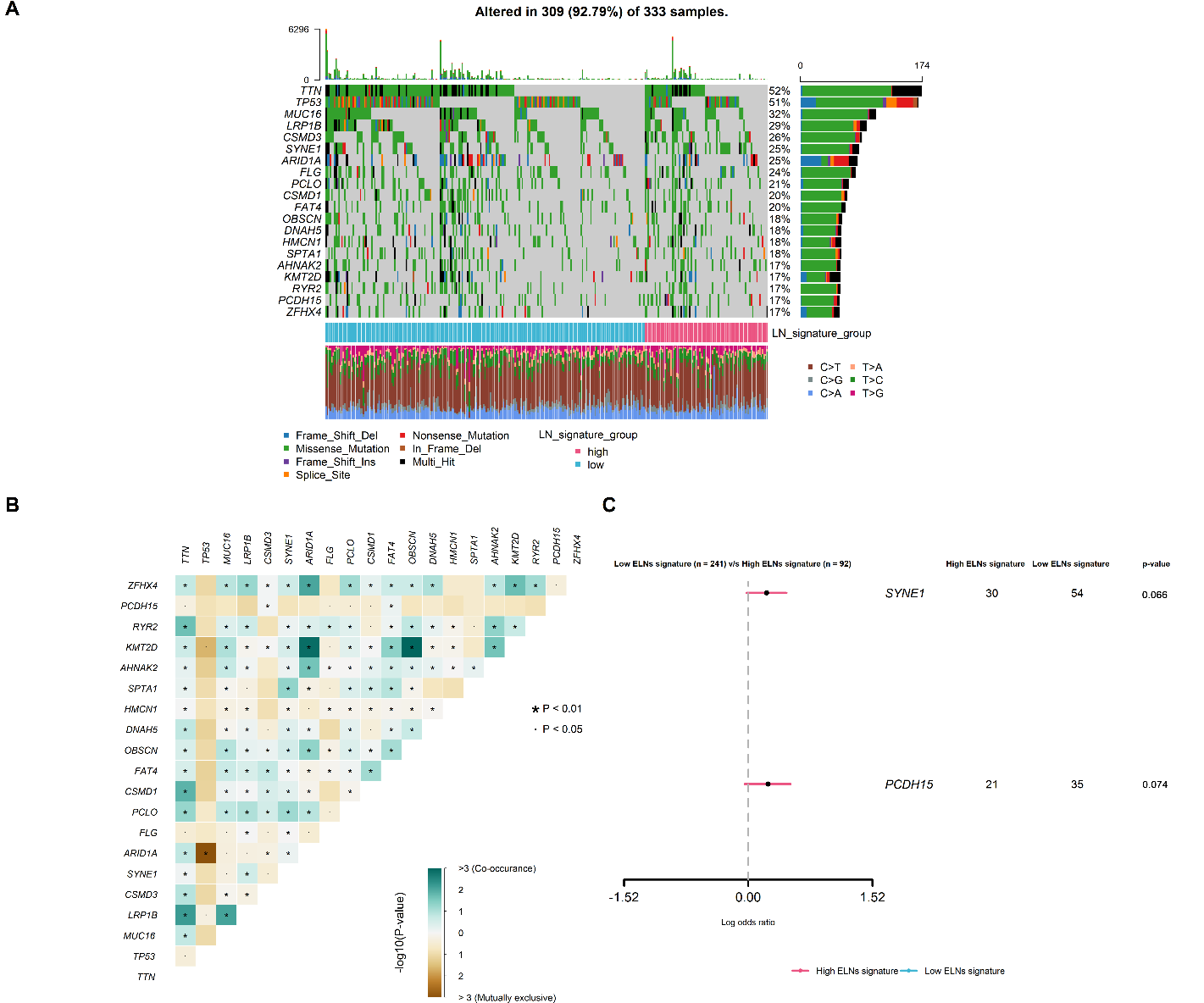


**FIGURE S9 |** Mutation patterns of GC patients. **(A)** The mutational patterns of 333 GC patients in the low- and the high- ELN signature groups displayed by the oncoplot. **(B)** Interaction effect of genes mutating differentially in patients in the low- and the high- ELN signature groups. **(C)** Forest plot of genes mutating differentially in patients of the low- and the high-ELN signature groups.


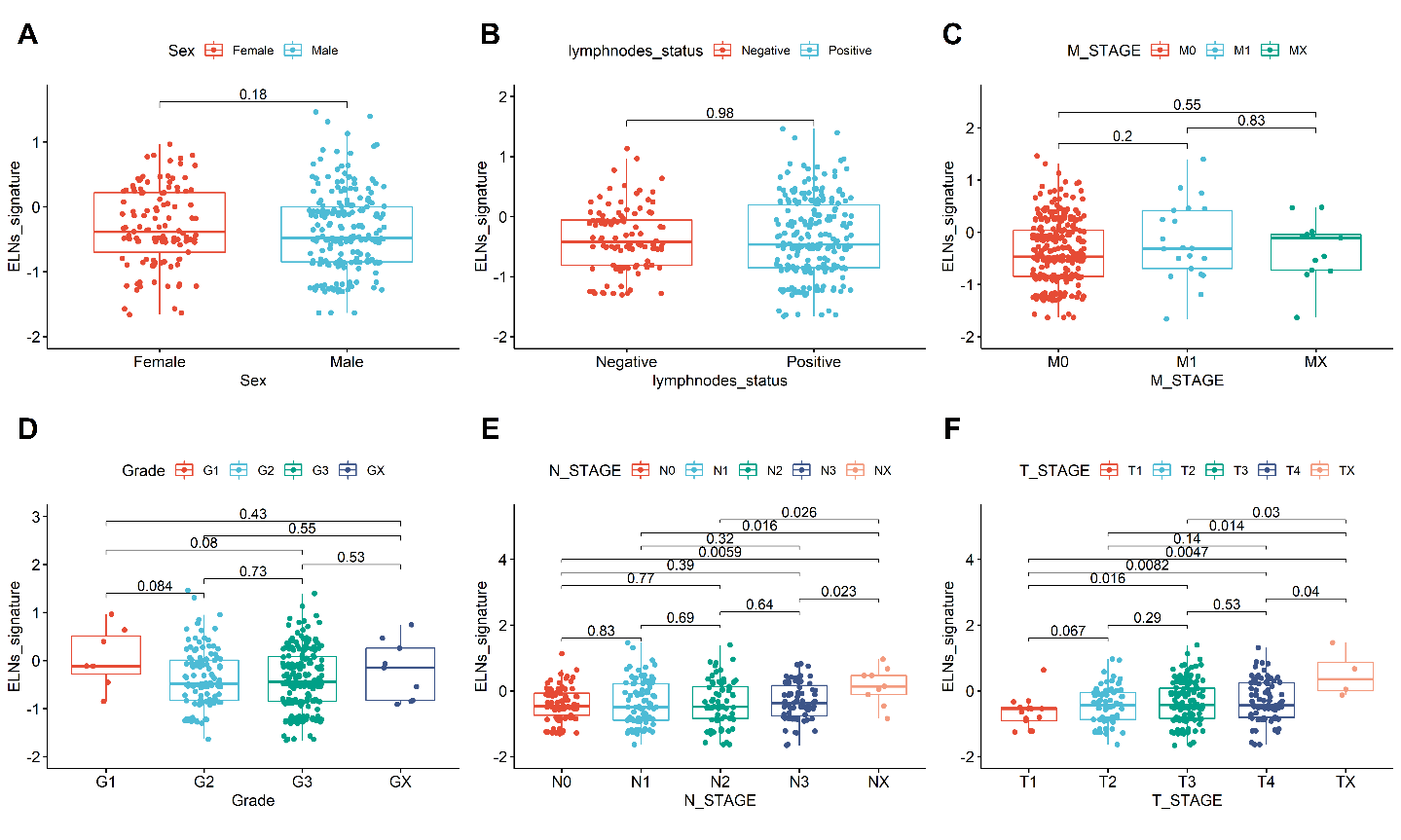


**FIGURE S10 |** Stratified analysis of clinical characteristics for the ELNs signature. Wilcoxon rank-sum or Kruskal-Wallis rank-sum tests were used to assess statistical significance. **(A)** Sex. **(B)** Grade level. **(C)** N stage. **(D)** M stage. **(E)** Lymph nodes status. **(F)** stage.


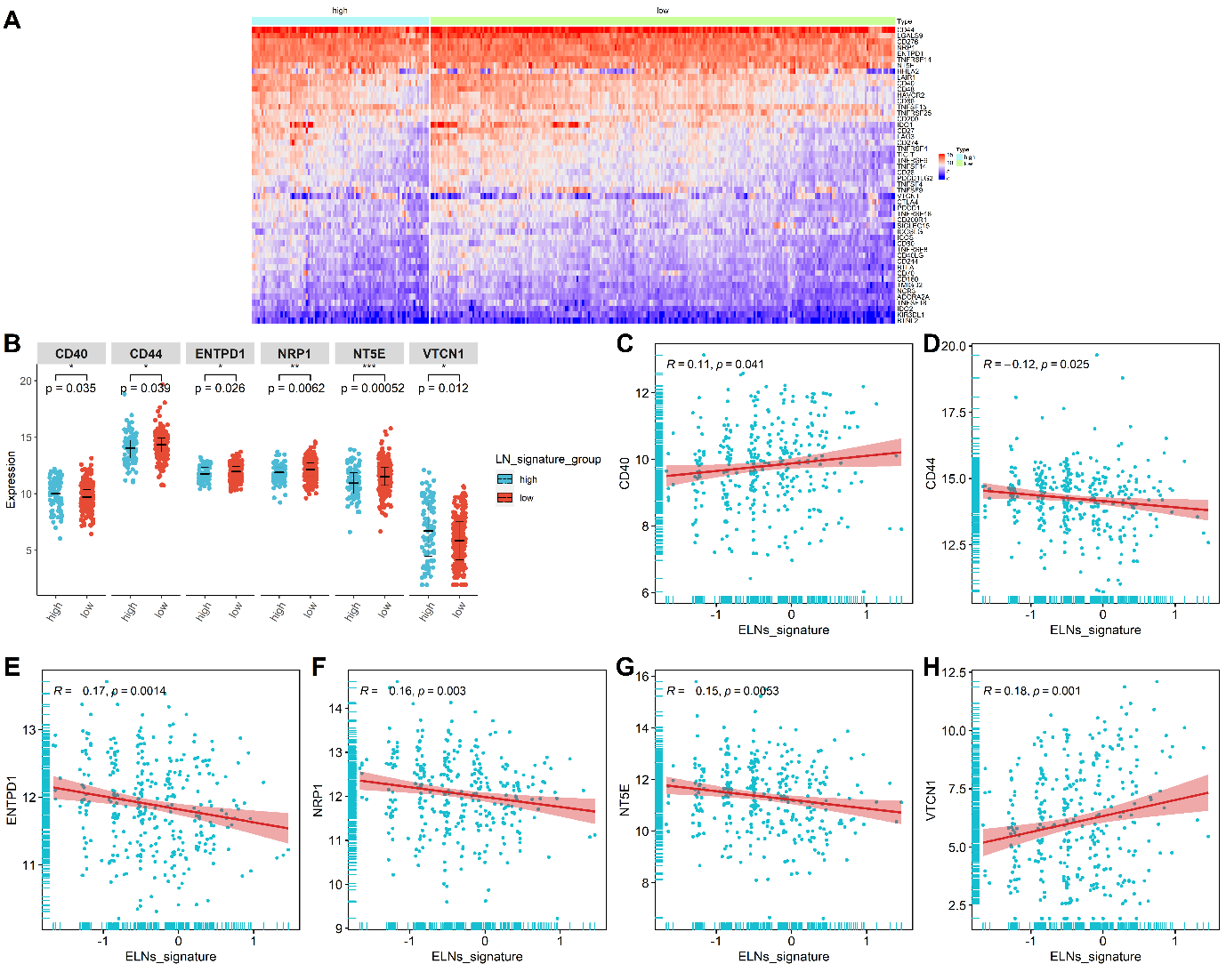


**FIGURE S11 |** Association between the ELNs signature and immune checkpoints genes. **(A)** Immune checkpoint heatmaps between low- and high-risk groups. **(B)** CD40, CD44, ENTPD1, NRP1, NT5E, and VTCN expression in two groups (Wilcoxon rank-sum test). **(C-H)** Correlation between the ELNs signature and the expression of (C) CD40, (D) CD44, (E) ENTPD1, (F) NRP, (G) NT5E, and (H) VTCN1 (Pearson correlation analysis).
