# Supplementary figures and images for: A nomogram model based on the number of examined lymph nodes–related signature to predict prognosis and guide clinical therapy in gastric cancer
Source: Front Immunol. 2022 Nov 2;13:947802. doi: 10.3389/fimmu.2022.947802 (PMC9667298; doi:10.3389/fimmu.2022.947802)

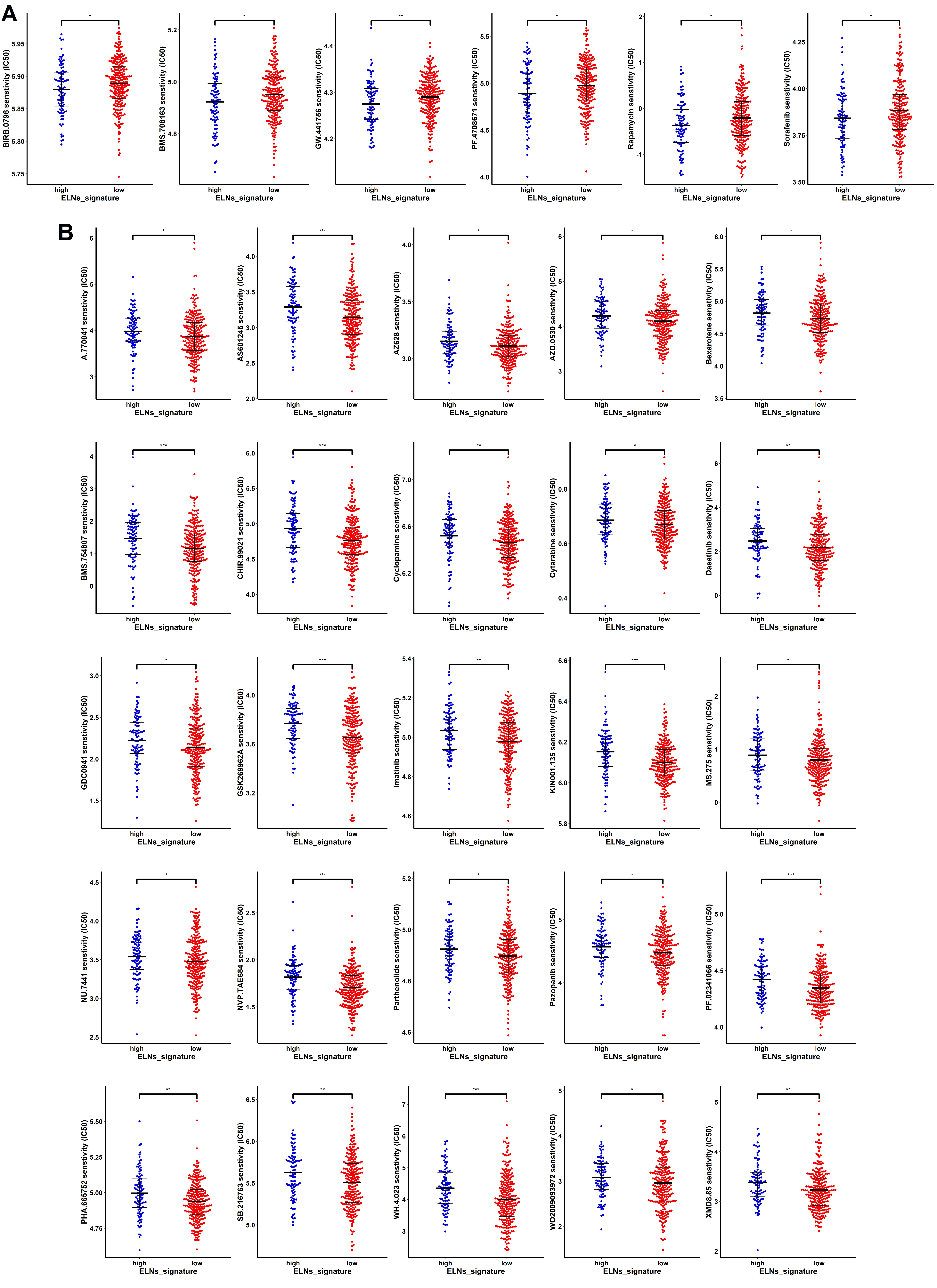

Supplement: Supplementary file 2 [file DataSheet_2.zip › Data Sheet 2/Supplementary Image/FigureS13.tiff]
